# Congruency of color–sound crossmodal correspondence interacts with color and sound discrimination depending on color category

**DOI:** 10.1177/20416695231196835

**Published:** 2023-08-30

**Authors:** Kenta Miyamoto, Yuma Taniyama, Kyoko Hine, Shigeki Nakauchi

**Affiliations:** 13129 Toyohashi University of Technology, Japan

**Keywords:** crossmodal correspondence, Stroop effect, audiovisual integration

## Abstract

People occasionally associate color (e.g., hue) with sound (e.g., pitch). Previous studies have reported color–sound associations, which are examples of crossmodal correspondences. However, the association between both semantic and perceptual factors with color/sound discrimination in crossmodal correspondence remains unclear. To clarify this, three psychological experiments were conducted, where Stroop tasks were used to assess automatic process on the association. We focused on the crossmodal correspondence between color (Experiment 1)/color word (Experiment 2) and sound. Participants discriminated the color/word or the sound presented simultaneously. The results showed the color–sound bidirectional enhancement/interference of the response by certain associations of the crossmodal correspondence (blue-drop and yellow-shiny) in both experiments. These results suggest that these Stroop effects were caused by the semantic factor (color category) and the perceptual factor (color appearance) was not necessary for the current results. In Experiment 3, response modulation by color labeling was investigated to clarify the influence of subjective labeling. Participants labeled a presented ambiguous color, which was a hue specification between two specific colors, by listening to the sound. The results revealed that the Stroop effect was caused only when the presented color was classified as the color related to the presented sound. This showed that subjective labeling played a role in the regulation of the effect of crossmodal correspondences. These findings should contribute to the explanation of crossmodal correspondences through semantic mediation.

Colors are not only used to describe the spectral variation of visible light, but they are also often used to express sound characteristics, as in the Japanese word “Kiiroi Seien” (yellow cheering), which refers to women’s high-pitched cheering voice. Expressions that associate color with sound are used not only in Japanese but also in other languages, such as German and Spanish (Ludwig et al., 2011), thereby suggesting that this association extends beyond a specific language or culture. This association has been studied as crossmodal correspondence, a phenomenon that has recently drawn a lot of interest. Crossmodal correspondences are sometimes identified between seemingly unrelated and surprising attributes, thereby reflecting the underlying influence of cultural differences on human constructs.

## Crossmodal Correspondences

Humans occasionally perceive the environment, whereby different sensory information is complementarily combined, in the so-called crossmodal correspondences (Gallace & Spence, 2006; Hamilton-Fletcher et al., 2017; Marks, 1987; Spence, 2011). Crossmodal correspondence refers to a compatibility effect between attributes or dimensions of a stimulus in different sensory modalities (Spence, 2011). Crossmodal correspondence could result in the modulation of actual experiences (e.g., red-colored white wine can be perceived as having the odor of red wine) (Morrot et al., 2001) and perceptual linkages between attributes (e.g., lemons are linked with fast more than slow) (Woods et al., 2013). Moreover, these crossmodal correspondences are expected to improve multisensory experiences in human–computer interaction (Lin et al., 2021; Metatla et al., 2019). Therefore, it is important to understand the way in which the human brain integrates multisensory information to realize higher levels of realism and engagement.

Spence (2011) suggested three categories of crossmodal correspondence based on its nature: structural, statistical, and semantic. Structural crossmodal correspondence is based on a shared neural representation used by different stimulus modalities to code (Spence, 2020). The correspondence between the loudness of sound and the brightness of light is classified in this category, and it is considered a relationship based on the magnitude of the stimulus between hearing and vision. Statistical crossmodal correspondence is an association based on patterns constructed from experience (Baier et al., 2006). The correspondence between the pitch of a sound and the size of the emitting object is classified in this category. In nature, the smaller the object, the higher the pitch, and the larger the object, the lower it sounds, which is corroborated by empirical statistics. Semantic correspondence is formed through linguistic terms (Gallace & Spence, 2006; Martino & Marks, 1999). Since equivalent expressions are used for sound pitch and spatial height, an association is formed between sound frequency and spatial height in the process of language acquisition. Regarding semantic crossmodal correspondence, Walker (2012) insisted that it was more suitable for calling lexical correspondences. This lexical relevance of the stimulus affected the increased discrimination performance (Melara & Marks, 1990). These classifications have not achieved a high degree of consensus, and in some cases, such as the crossmodal correspondence between color and sound, none of these categories are applicable (Spence, 2011). In addition to these three categories, emotionally mediated (hedonic) correspondence has been recently discussed. Emotionally mediated correspondence is formed based on the emotional or affective association of component stimuli (Spence, 2011). Regarding emotionally mediated correspondence, Palmer et al. (2013) showed the crossmodal correspondence between music and colors and insisted that this association was mediated by common emotional associations.

### Color–Sound Crossmodal Correspondences

Color–sound crossmodal correspondence has been discussed for a long time, and it is increasingly being studied (Spence & Di Stefano, 2022: see as review). Marks (1987) investigated the association between the color and brightness of visual stimuli and the pitch of auditory stimuli using a discrimination task and reported that brighter colors were associated with higher pitches. Hamilton-Fletcher et al. (2017) asked the experiment’s participants to adjust an equiluminant color to stimuli containing only a specific frequency of sound. Pitch was found to have a crossmodal correspondence to hue, with lower sound frequencies reported to correspond to blue and higher frequencies to yellow. Similar experiments have been conducted, both on synesthetes, who perceive colors when they hear sounds, and nonsynesthetes. Ward et al. (2006) investigated the difference in the pairing of colors and sounds between synesthetes and nonsynesthetes, where participants were required to answer the color that they associated most strongly with the presented sound, and both synesthetes and nonsynesthetes reported darker colors for the low-pitched sounds and brighter colors for the high-pitched sounds.

Some studies used more complex stimuli for color–sound correspondence (Barbiere et al., 2007; Palmer et al., 2013). In a study conducted by Palmer et al. (2013), the participants were asked to listen to 18 classical pieces by Bach, Brahms, and Mozart, and then select the five optimal and least optimized matches from a color patch. Their results showed a strong correlation between color saturation, lightness and tempo, and cold/warm colors and song tunes. Moreover, the assessment of the correlations between color/sound stimuli and emotional evaluations yielded robust significant results. This confirmed the presence of a crossmodal correspondence between color and music, which was complex stimuli and also suggested that this correspondence was based on emotion as well as sensory experience.

Such crossmodal correspondences could also affect actual perceptual performance. Sun et al. (2018) reported that response speed for the pair-discrimination task was affected in implicit rather than explicit matching. In their study, participants were asked to explicitly memorize crossmodal correspondence between color and sounds. The participants were then asked to respond to whether the presented pairs of color and sound were the same as the memorized pairs. The participants who memorized congruent pairs of color and sound responded more quickly and accurately than those who memorized the incongruent pairs. Other studies reported that crossmodal correspondences influenced the participants’ responses based on crossmodal congruency in the speeded discrimination task (Anikin & Johansson, 2019; Ho et al., 2014; Sun et al., 2018), whereas there were studies that reported no effects of configuring crossmodal correspondences (Bernstein et al., 1971; Ward et al., 2006). In a study conducted by Bernstein et al. (1971), the participants were presented with a color patch and a sound stimulus at the same time. They discriminated on the colors, and the response time did not depend on the color patch or the sound frequency. In a study conducted by Ward et al. (2006), Stroop interference occurred among synesthetes, but not among nonsynesthetes, in their responses to pairs created by matching color and sound.

As previously described, the existence of crossmodal correspondence between color, especially hue, and sound, including pure tone and music, has been reported. However, it remains unclear whether there is crossmodal correspondence between hue and environmental sound. In daily life, we experience a lot of sound in several situations. To reveal the role of color-sound correspondence among humans, it is important to assess the way in which the relationship between hue and sound generated in our environment is involved in perceptual performance.

### Dependence of the Semantic or Perceptual on Crossmodal Correspondences

As mentioned previously, there have been several studies on crossmodal correspondence, and many links between different sensory modalities have been reported. However, it is not clear whether crossmodal correspondence depends on semantic or perceptual processing. One explanation that supports the involvement of semantic processing is the mediation of the verbal codes (Gallace & Spence, 2006). Gallace and Spence stated that crossmodal correspondence was formed by having common words, such as high-pitched sound and high location. In other words, semantic processing was involved in crossmodal correspondences. The semantic mediation of crossmodal correspondences has been discussed, and certain studies reported the involvement of semantic processing (Gallace & Spence, 2006; Martino & Marks, 1999). Martino & Marks (1999) found that the congruency effect was observed when using black and white color patches and lightness-related words (e.g., night and day) as the visual stimuli. This indicated that the words’ meanings caused the congruency effect through the lightness-pitch crossmodal correspondence. Conversely, indirect lightness-related words (e.g., bad and good) did not produce the congruency effect in their study. Therefore, the congruency effect is considered to have been caused only by the direct meaning of the words. Moreover, Gallace & Spence (2006) found that the congruency effect was observed in the crossmodal correspondences between size and pitch. The study examined the modulation of size perception by pitch and found that it was not modulated, thereby indicating that the congruency effect was caused by the semantic and not by the perceptual factor. This semantic mediation was reported for the color–haptic and color–odor crossmodal correspondences (Slobodenyuk et al., 2015; Spence, 2020; Stevenson et al., 2012). Those studies supported the semantic dependence of the crossmodal correspondences.

Although several studies (Gallace & Spence, 2006; Martino & Marks, 1999; Slobodenyuk et al., 2015; Spence, 2020) strongly suggested that semantic processing was involved in crossmodal correspondences, one study suggested that perceptual processing was involved in crossmodal correspondence (Maeda et al., 2004). The study investigated the modulation of visual motion by pitch. The participants were required to respond to the visual motion of gratings with ambiguous motion while listening to ascending or descending pitched sounds. The results revealed the perceptual modulation of visual motion by pitch, as presenting the words “up” or “down” did not cause this effect. This result could be explained by perceptual mediation rather than semantic mediation. Moreover, from the perspective of neural processing, it was suggested that both visual/auditory regions and higher-level regions were involved in crossmodal correspondence (Sadaghiani et al., 2009). As discussed above, it remains unclear whether crossmodal correspondences depend on semantic or perceptual mediation or both.

### Quantitative and Qualitative Characteristics of Stimuli

The quantitative or qualitative characteristics of stimuli that contribute to crossmodal correspondence have also been discussed. Certain studies focused on the prothetic and metathetic dimensions as the distinctions of a stimulus based on its characteristics (Spence & Di Stefano, 2022; Stevens & Galanter, 1957). Prothetic continua are described as stimuli with quantitative characteristics and a clear more than and less than relationship. Brightness of color and loudness of sound are categorized as prothetic dimensions as they are expressed as brighter/darker or louder/quieter. Conversely, metathetic continua are stimuli with qualitative and categorical characteristics expressed as position or type of relationship. Hue perception on color and pitch perception on sound are categorized as metathetic dimensions as they are expressed as color or sound pitch. If hue naming is affected in the context of crossmodal correspondence, qualitative characteristics of stimuli could be involved in crossmodal correspondence. Therefore, hue–sound crossmodal correspondence is one of the methods for assessing the contribution of qualitative characteristics in visual stimuli.

## Study Objective

This study aims to clarify the way in which semantic and perceptual processing relate to color–sound crossmodal correspondence, particularly the effect of hue and environmental sound on perceptual performance. In this study, we focused on hue to clarify whether quantitative characteristics of stimuli were involved in environmental correspondence. We also took note of environmental sounds because it was unclear whether there were crossmodal correspondences between hue and environmental sounds. If there is a crossmodal correspondence between different sensory modalities, the response to the stimulus should be affected by processing for another stimulus. The degree of crossmodal correspondences could be assessed by comparing the magnitude of interference. To investigate the interference caused by crossmodal association, we used the Stroop task, which can implicitly compare the effects of the stimuli’s congruency.

Stroop interference is a psychological phenomenon reported by John Ridley Stroop in 1935 (Stroop, 1935). It occurs when the name of a color is presented in letters of the same color (e.g., the word blue written in blue ink) and when the color name is not the same as the color of the ink with which it is written (e.g., the word red written in blue ink). The relative response time to identify the ink color is delayed in the latter case (Stroop, 1935).

Stroop interference is used to assess the ability to suppress the cognitive interference that occurs when the processing of a stimulus affects the parallel processing of another attribute of the same stimulus (Scarpina & Tagini, 2017). One of the explanations, based on the parallel distributed processing model, suggests that owing to training, the processing of letter reading has a higher intensity than that of color naming, and this difference in intensity causes interference (Braem et al., 2019; Cohen et al., 1990). Other studies report the semantic Stroop effect where the color-associated words, such as “sky” interfere with color-naming as well as color-name wording, thereby indicating that Stroop effect is caused by a semantic conflict (Kinoshita et al., 2018; Klein, 1964; Risko et al., 2006). This study is based on the hypothesis that if the Stroop effect is caused by conflict of the semantic information between words and colors, then interference should be observed as the conflict of the crossmodal correspondences between colors and sounds (Klein, 1964). Previous research on audiovisual crossmodal correspondences reported that congruency of the crossmodal correspondences improved the responses in the discrimination task (Marks, 1987). In this study, we investigated the Stroop interference of the color–sound crossmodal correspondences using color patches and words representing the colors, in line with previous studies (Gallace & Spence, 2006; Maeda et al., 2004; Martino & Marks, 1999).

In Experiment 1, we assessed the Stroop interference using crossmodal correspondence for color–sound association. To assess the effect of environmental sound, sound stimuli used in this study were selected from the International Affective Digitized Sound (IADS-E) database (Yang et al., 2018). As mentioned above, the Stroop effect is the phenomenon whereby the proportion of correct responses and/or response time is interfered with owing to the conflict of word meaning and color processing. If the two stimuli are associated with each other, the proportion of correct responses and/or response time will interact with the congruency of the crossmodal correspondences.

In Experiment 2, the crossmodal Stroop task involving color name stimuli, which did not contain the perceptual factor (color appearance), was conducted. Experiment 2 was conducted based on the hypothesis that Stroop interference depends on the semantic factor (color category) as humans categorize colors when making color judgments. Since a typical Stroop effect is caused by the conflict of color information, the conflict of crossmodal correspondence and not the color appearance is crucial to causing the Stroop effect. Therefore, we hypothesized that the crossmodal Stroop effect also occurred when the color word instead of the color patch was used as the visual stimulus.

In Experiment 3, we investigated the impact of subjective color labeling by manipulating the physical color appearance and requiring participants to respond with the color category of the ambiguous colored patches. Kubat et al. (2009) reported that, when categorizing words and shapes associated with colors presented in intermediate colors, the color categorization judgment was biased toward the associated color. To assess whether qualitative characteristics of color stimuli affected the color–sound crossmodal correspondence, the point of subjective equality (PSE) for the color category boundary and the reaction time were analyzed.

## Experiment 1. Effects of Color–Sound Crossmodal Stroop

The purpose of this experiment was to examine the hypothesis that crossmodal correspondences affect classification processing in the color and sound discrimination task. This was achieved by investigating the interference in the associations between color and sound using the Stroop paradigm. In an experiment conducted by Bernstein et al. (1971), which investigated the interference effect between color and sound, participants were asked to distinguish between blue and red patches while listening to low and high pitch sounds. The results showed no interaction between the colors and sounds. In addition, Ward et al. (2006) investigated the Stroop interference between colors and sounds and reported no interference among nonsynesthetes, whereas an interference was observed among synesthetes. More recent studies revealed that color–sound crossmodal correspondences were generated for blue and yellow (Hamilton-Fletcher et al., 2017; Palmer et al., 2013). Although certain studies investigated the relationship between color and sound, it remains unclear whether there is crossmodal correspondence between color and environmental sound. In this study, we used sound stimuli selected from the IADS-E database (Yang et al., 2018), which comprises natural sounds common in daily life. To assess the interference effects, blue and yellow color patches were simultaneously presented with a low-pitched falling object sound and a high-pitched bell tone from the IADS-E, which were considered to be associated with each color (Anikin & Johansson, 2019; Sun et al., 2018; Ward et al., 2006), and the proportions of correct responses and *z*-scored response time were analyzed.

### Materials and Methods

#### Participants

A power analysis of repeated-measures analysis of variance with G*Powers (Faul et al., 2009) recommended the enrollment of 20 participants corresponding to an effect size *f* of 0.24, power of 0.80, *p* value of .05, one group, six measurements, correlation of 0.5 among repeated measures, and a nonsphericity correction ϵ of 1. Therefore, we enrolled 20 adult participants. Twenty adult males (age range: 22–27 years (mean (*M*) = 23.1, standard deviation (*SD*) = 1.4)) with appropriate blue and yellow discrimination ability or corrected vision and normal hearing, participated in the experiments. All participants were provided with the procedural details. Informed consent was obtained from all the participants. The experiment was conducted after receiving approval from the Ethical Review Committee for Research Involving Human Subjects of Toyohashi University of Technology (Approval number: H31-01).

#### Stimuli and Apparatus

Four color patches were used for the visual stimuli: blue, yellow, dark gray (the luminance was approximately the same as that of blue), and light gray (the luminance was approximately the same as that of yellow). The luminance of the four stimuli was controlled such that the Y-value difference from the background was almost equal. The background was gray with a lightness that was approximately the middle point of the Y-value, between blue and yellow. The two gray colors were selected as the stimuli for the control condition to analyze whether the experiments’ results were due to hue or luminance. Table 1 shows the coordinates of the color stimuli.

**Table 1.** Coordination of color stimuli (Experiment 1).

Session		RGB			XYZ	
Blue	0.0000	0.0000	1.0000	0.1804	0.0722	0.9503
Yellow	1.0000	0.9333	0.1412	0.7213	0.8253	0.1380
Dark gray	0.2941	0.2941	0.2941	0.0669	0.0704	0.0766
Light gray	0.9216	0.9216	0.9216	0.7897	0.8308	0.9046
Background	0.7059	0.7059	0.7059	0.4338	0.4564	0.4970

These were determined to have approximately equal Y-value differences from the value of the background. In Experiment 1, the RGB-values were used to present the color stimuli. The values of XYZ were calculated from the value of RGB on the left columns.

The auditory stimuli were selected from the IADS-E data set (Yang et al., 2018): drop, a low-pitched falling object sound and shiny, a high-pitched shimmering bell tone. These sounds were supposed to be associated with blue and yellow, respectively (Hamilton-Fletcher et al., 2017; Palmer et al., 2013; Ward et al., 2006). The loudness of these sound stimuli was adjusted to 80 phon using the free software MP3Gain. The loudness affected the color matching in a previous study(Sun et al., 2018). We determined the appropriate volume level as 80 phon from the participants' interviews in a preliminary experiment.

The experiment was conducted in a dark room and run in MATLAB 2019b (MathWorks, Natick, MA, USA) using Psychtoolbox-3 (Kleiner et al., 2007). The instructions of the experiment and visual stimuli were presented on a calibrated liquid crystal display (LCD) monitor (Display++, Cambridge Research Systems Ltd) with a resolution of 1920 × 1080 pixels and a refresh rate of 120 Hz. The auditory stimuli were presented via headphones (MDR-M 1ST, Sony). While participants observed the stimulus, each participant was seated on a chair facing the display, with their heads secured on a chin rest to maintain a constant distance (60 cm).

#### Procedure

At the beginning of the experiment, the auditory stimuli were presented to confirm that they played correctly to the participants. After the participants listened to both auditory stimuli, the instructions for the experimental procedure were presented on the screen along with the task description, and the participants were informed which of the two tasks (sound discrimination task/color discrimination task) was about to start. After the button was pressed, a fixed cross was presented for 1,000 ms. The auditory stimulus was then presented, and a visual stimulus was presented after 40 ms in random order. To match the difficulty between the sound and color discrimination tasks for the reaction time, we checked the reaction time when dark/light gray was presented as the color stimulus in both tasks in a preliminary experiment. In our preliminary experiment, there was no difference between the reaction times for the sound and color discrimination tasks when the auditory stimulus was presented 40 ms before the presentation of a visual stimulus. Therefore, we decided to present the auditory stimulus 40 ms before the presentation of the visual stimulus. In the sound discrimination task, participants were required to indicate the sound (drop/shiny) while they were asked to indicate the color (blue/yellow) in the color discrimination task. The sound and color stimuli were simultaneously removed after confirming the participant’s response.

**Figure 1.**
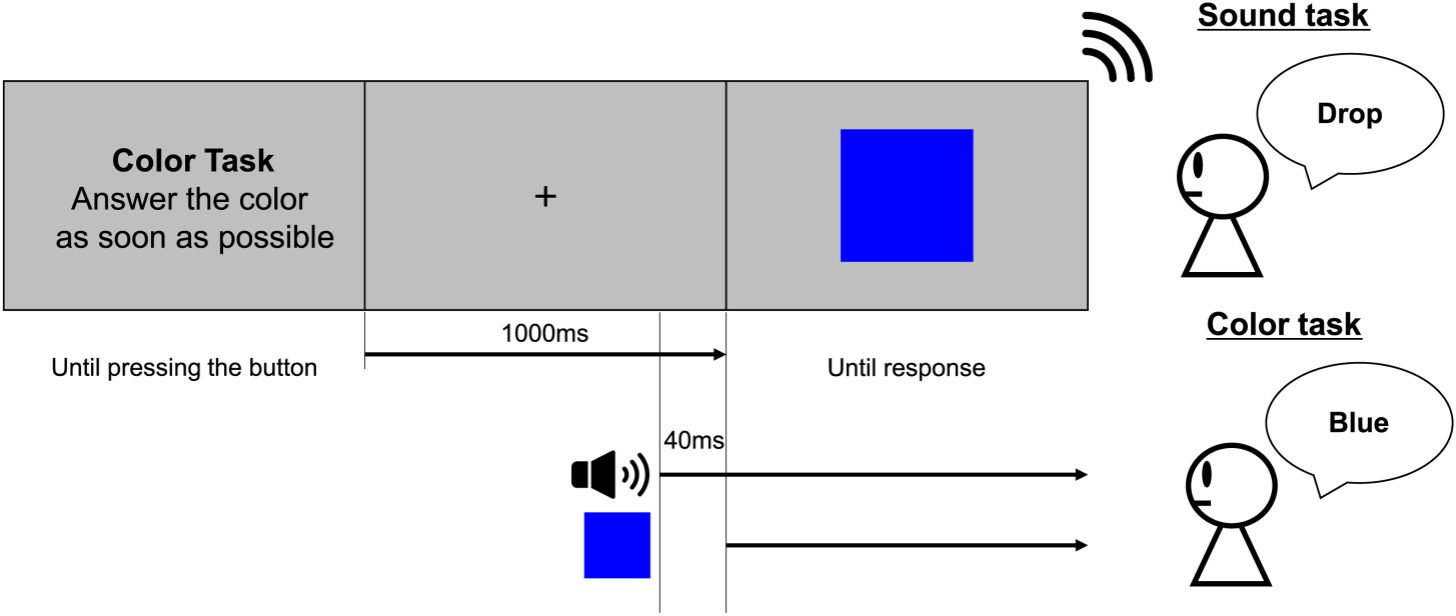
Experimental protocol for one trial (Experiment 1). There were two types of tasks in the experiment: sound and color discrimination tasks. The participant was required to identify the sound or color presented after the 1,000 ms fixation by pressing the button. The sound stimulus was presented 40 ms before the color stimulus. Both stimuli were presented until the participant’s response was confirmed.

During the sound discrimination task, drop or shiny was presented, and one of four types of color stimuli (blue/yellow/dark gray/light gray) was presented as 2×4=8 conditions. In Experiment 1, two gray colors were used to compare the difference between hue and luminance. In the color discrimination task, there were two types of color stimuli (blue/yellow) and three types of sound stimuli (drop/shiny/none), accounting for 2×3=6 conditions. Each participant performed (8 conditions + 6 conditions) × 20 repetitions = 280 trials. The sound and color discrimination tasks were alternated every six to eight trials. Each sound and color stimulus was presented in random order. To eliminate the influence of the button position, the placement of the buttons was randomized for each participant, and they were required to press the button using both thumbs. Figure 1 shows an example of the flow of one trial.

#### Analysis

MATLAB R2018b (MathWorks) and R software (Version 4.2.0) were used for the analyses. The trials in the sound discrimination task were separated into four groups based on congruency (congruent/incongruent) and type of color (chromatic/achromatic). The trials in the color discrimination task were separated into three groups (congruent/incongruent/control) based on the combination of color and sound stimuli (Table 2).

**Table 2.** Combination of stimuli and congruency conditions (Experiment 1).

Task	Sound	Color	Congruency	Type of color
Sound	Drop	Blue	Congruent	Chromatic
	Shiny	Blue	Incongruent	Chromatic
	Drop	Yellow	Incongruent	Chromatic
	Shiny	Yellow	Congruent	Chromatic
	Drop	Dark gray	Congruent	Achromatic
	Shiny	Dark gray	Incongruent	Achromatic
	Drop	Light gray	Incongruent	Achromatic
	Shiny	Light gray	Congruent	Achromatic
Color	Drop	Blue	Congruent	—
	Shiny	Blue	Incongruent	—
	None	Blue	Control	—
	Drop	Yellow	Incongruent	—
	Shiny	Yellow	Congruent	—
	None	Yellow	Control	—

These combinations were based on the previous studies on color–sound crossmodal correspondence (Hamilton-Fletcher et al., 2017; Palmer et al., 2013; Ward et al., 2006).

The proportions of correct responses and the average response time of each condition were calculated for each participant. A previous study recommended the use of reaction time with the *z*-score to account for the difference of the individual processing speed (Hedge et al., 2018). Therefore, we used reaction time with the *z*-score in this study.

An analysis of variance (ANOVA) was then performed on the distribution of the proportions of correct responses and response time for all participants. ANOVA, a function that runs on the free statistical software R, was used for ANOVA.

### Results

#### The Proportions of Correct Responses

**Figure 2.**
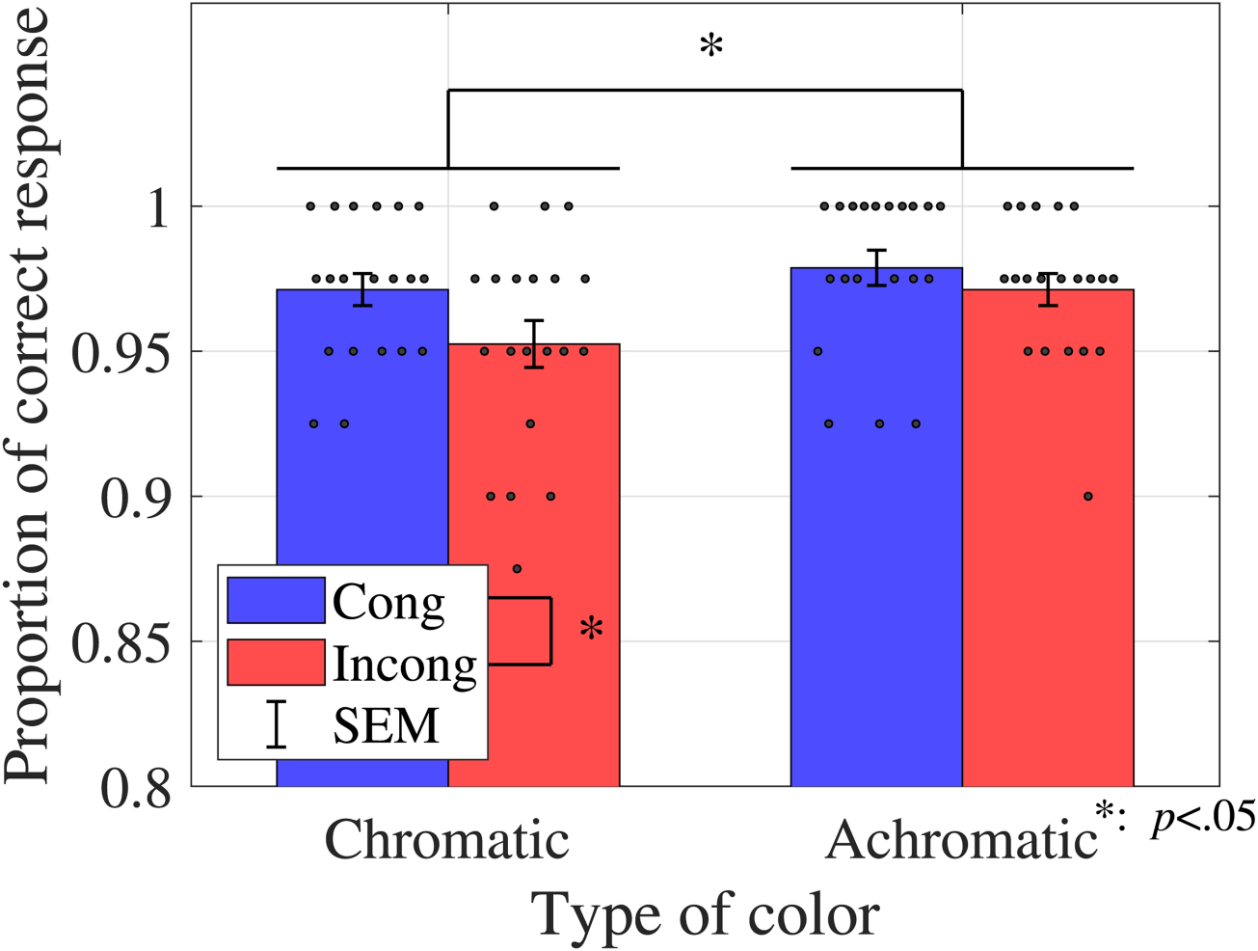
Proportions of correct responses (sound discrimination task in Experiment 1). Bar graphs and error bars show the average and standard errors of proportion of the correct responses, respectively. Dots show the result for individuals (*N* = 20). The trials were separated based on the congruency and type of color conditions (see Table 2). Higher accuracies on the congruent condition and the achromatic condition were observed. Abbreviations: Cong = congruent; Incong = incongruent; SEM = ±1 standard error of the mean.

**Figure 3.**
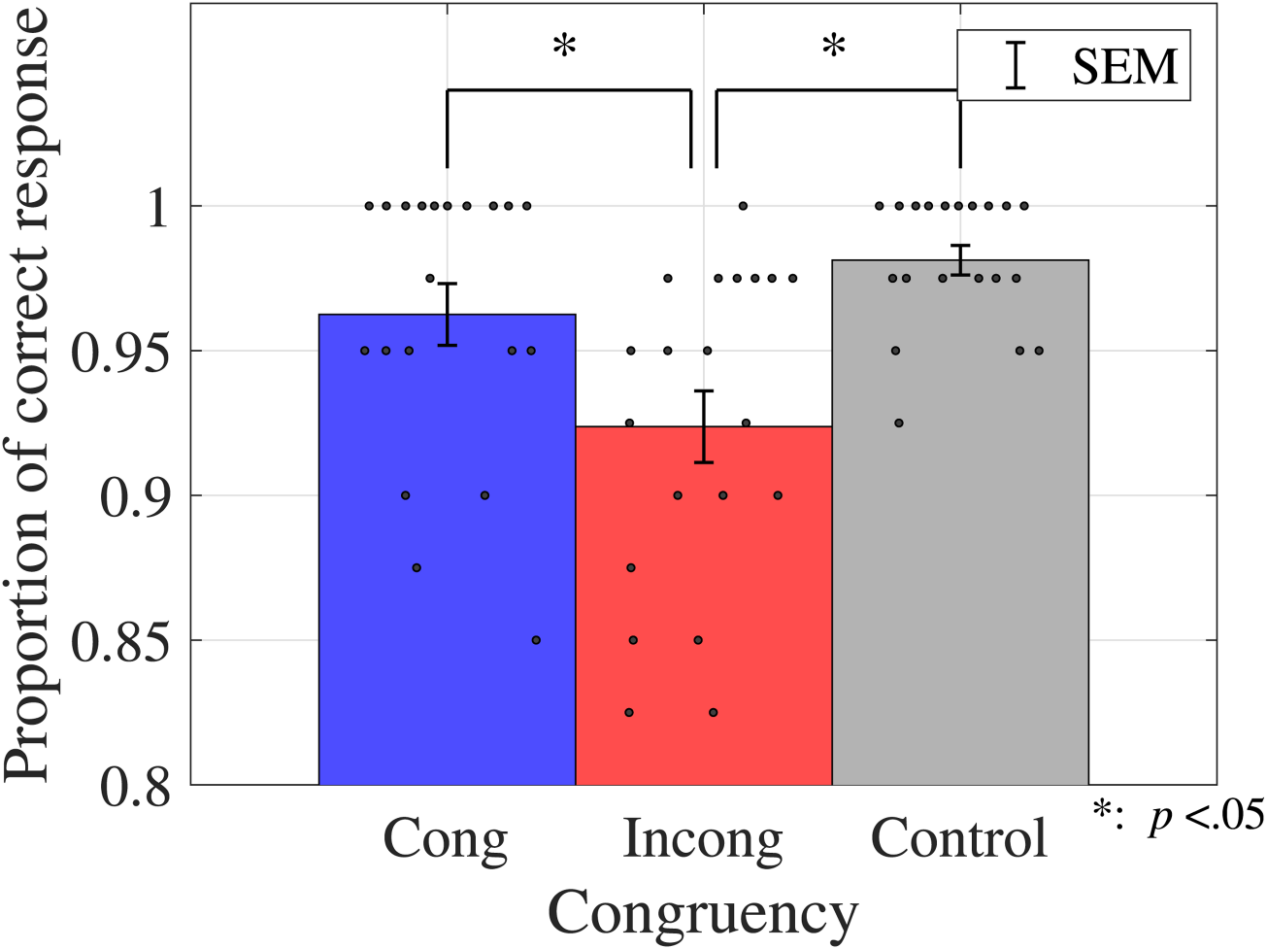
Proportions of correct responses (color discrimination task in Experiment 1). Bar graphs and error bars show the average and standard errors of proportion of the correct responses, respectively. Dots show the result for individuals (*N* = 20). The accuracy in the incongruent condition was lower than that in the other two conditions. Abbreviations: Cong = congruent; Incong = incongruent; SEM = ±1 standard error of the mean.

The proportions of correct responses were calculated for each condition. They are shown in Figure 2 (sound discrimination task) and in Figure 3 (color discrimination task). The error bars indicate the ±1 standard error of the mean (SEM).

Two-way ANOVA was performed to analyze the proportions of correct responses in the sound discrimination task, considering congruency, and type of color as factors. The results revealed the main effect of congruency ( $F(1,19)=6.6553,p=.0184,\eta_{p}^{2}=0.2594$ ) and type of color ( $F(1,19)=5.3065,p=.0327,\eta_{p}^{2}=0.2183$ ).

One-way ANOVA was performed to compare the effect of congruency on the proportions of correct responses in the color discrimination task. The results revealed a significant primary effect of congruency ( $F(1.8,34.23)=11.0105,\text{\textbackslash{},p}=.0003,\eta_{p}^{2}=0.3669$ ). The multicomparison revealed a significant difference between incongruent and control conditions ( t(19)=4.1955,p=.0005,adj.p=.0015 ) and between congruent and incongruent conditions ( t(19)=4.6459,p=.0088,adj.p=.0088 ). Table 3 shows the results of the ANOVA for both the sound and color discrimination tasks.

**Table 3.** ANOVA results for the proportions of correct responses. In the sound discrimination task, high accuracy was observed for the congruent condition compared to the incongruent condition and on the achromatic condition compared to the chromatic condition. In the color discrimination task, low accuracy was observed for the incongruent condition.

Sound task		*F*	*p*		* $p.eta^{2}$ *
	Congruency	6.6553	.0184	*	0.2594
	Type of color	5.3065	.0327	*	0.2183
	Congruency × Type of color	0.7937	.3841	*ns*	0.0401
Color task		*F*	*p*		* $p.eta^{2}$ *
	Congruency	11.0105	.0003	***	0.3669
		*t*	*p*	*adj.p*	
	Incong < Control	4.1955	.0005	.0015	*
	Cong > Incong	4.6459	.0088	.0088	*
	Cong = Control	1.8311	.0828	.0828	*ns*

Abbreviations: Cong = congruent; Incong = incongruent; ANOVA = analysis of variance.

*: p<.05 , ***: p<.001 .

#### Response Time

**Figure 4.**
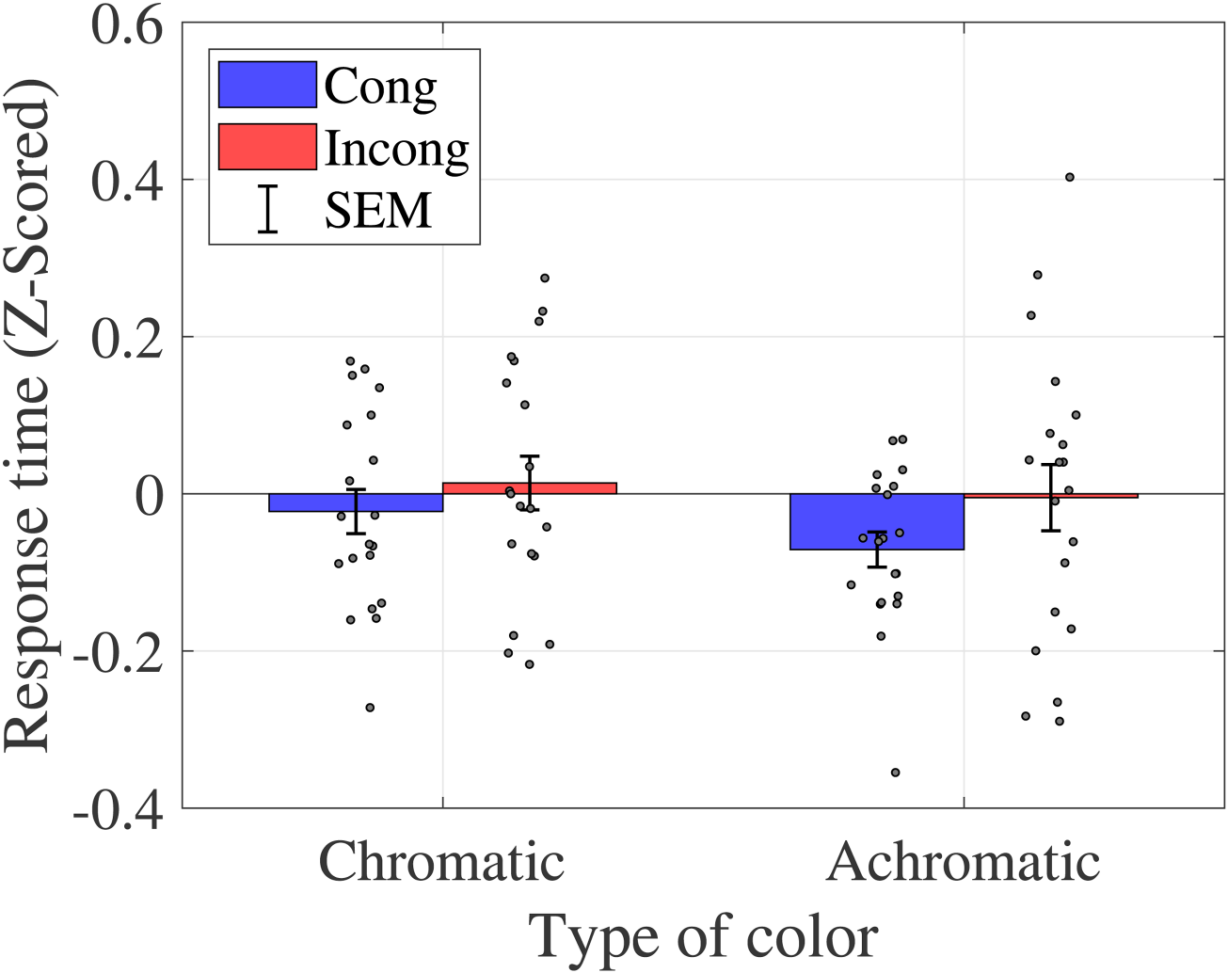
Average response time (sound discrimination task in Experiment 1). Bar graphs and error bars show the average and standard errors of response time, respectively. Dots show the results for individuals (*N* = 20). The trials were separated based on the congruency and type of color conditions (see Table 2). Significant differences were not observed among these conditions. Abbreviations: Cong = congruent; Incong = incongruent; SEM = ±1 standard error of the mean.

**Figure 5.**
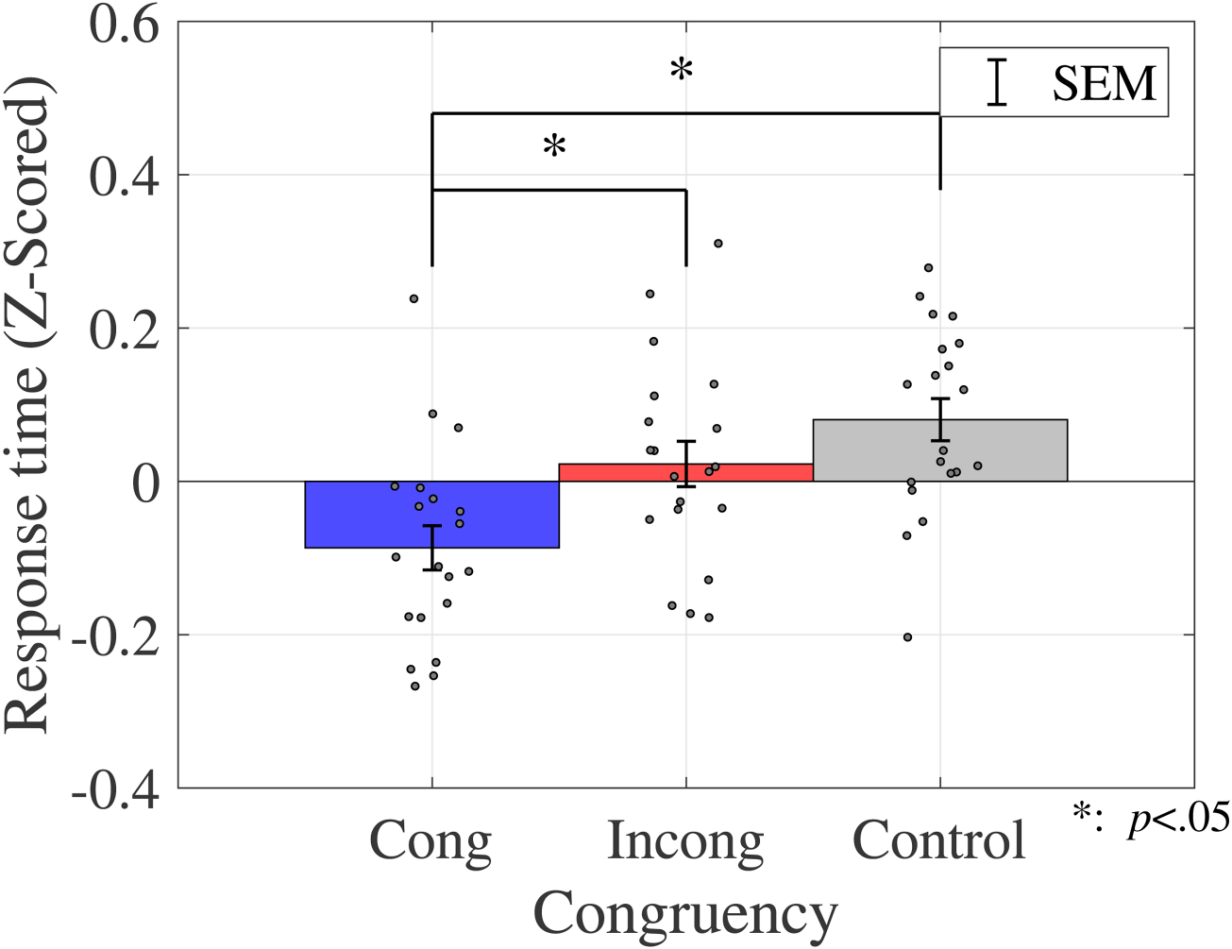
Average response time (color discrimination task in Experiment 1). Bar graphs and error bars show the average and standard errors of response time, respectively. Dots show the result for individuals (*N* = 20). The congruent condition had a significantly shorter response time than the other two conditions. Abbreviations: Cong = congruent; Incong = incongruent; SEM = ±1 standard error of the mean.

The average response time is shown in Figure 4 (sound discrimination task) and in Figure 5 (color discrimination task).

Similar to the analysis of the proportions of correct responses, a two-way ANOVA was performed to analyze the effect of crossmodal congruency and type of color on response time in the sound discrimination task (Table 4). The results reveal no statistically significant main effects of crossmodal congruency ( $F(1,19)=2.1511,p=.1588,\eta_{p}^{2}=0.1017$ ), type of color ( $F(1,19)=0.7697,p=.3913,\eta_{p}^{2}=0.0389$ ) or interaction between them ( $F(1,19)=0.1607,p=.6930,\eta_{p}^{2}=0.0084$ ).

**Table 4.** ANOVA results for the response time. In the sound discrimination task, no significant differences were observed between the conditions. In the color discrimination task, relatively shorter response time was observed in the congruent condition.

Sound task		*F*	*p*		* $p.eta^{2}$ *
	Congruency	2.1511	.1588	*ns*	0.1017
	Colored	0.7697	.3913	*ns*	0.0389
	Congruency × Colored	0.1607	.6930	*ns*	0.0084
Color task		*F*	*p*		* $p.eta^{2}$ *
	Congruency	6.2437	.0053	**	0.2473
		*t*	*p*	*adj.p*	
	Cong < Control	3.1962	.0048	.0143	*
	Cong < Incong	2.2455	.0368	.0368	*
	Incong = Control	1.3558	.1911	.1911	*ns*

Abbreviations: Cong = congruent; Incong = incongruent; ANOVA = analysis of variance.

*: p<.05 , **: p<.01 .

A one-way ANOVA was performed to assess the effect of crossmodal congruency on the response time in the color discrimination task (Table 4). The results revealed a significant main effect of the crossmodal congruency ( $F(1.9,36.08)=6.2437,p=.0053,\eta_{p}^{2}=0.2473$ ). The multicomparison revealed a significant difference between congruent and control conditions ( t(19)=3.1962,p=.0048,adj.p=.0143 ) and between congruent and incongruent conditions ( t(19)=2.2455,p=.0368,adj.p=.0368 ).

### Discussion

This experiment examined the hypothesis that crossmodal correspondences affected classification processing in sound and color discrimination tasks by investigating the interference with the Stroop paradigm in the associations between color and sound, particularly between hue and environmental sound. Although some previous studies reported no effect of configuring crossmodal correspondences (Bernstein et al., 1971; Ward et al., 2006), our results showed Stroop effects attributed to the congruency of crossmodal correspondences between color and environmental sound. This indicates that crossmodal correspondence between different sensory modalities was automatically involved in discrimination. In this study, the stimulus selection was based on the color–sound crossmodal research studies (Marks, 1987; Simpson et al., 1956; Wicker, 1968). In these studies, low-pitched sounds were associated with dark and blue colors (Simpson et al., 1956), whereas high-pitched sounds were associated with light (Marks, 1987; Wicker, 1968) and yellow colors. In addition, Hamilton-Fletcher et al. (2017) showed that low-pitched sounds were associated with the blue hue when properly controlling for lightness. These correspondences were consistent with the results of this study, where responses interacted with both hue and luminance in the sound discrimination task.

Notably, yellow is generally lighter than blue. Therefore, it is not obvious which of the two, hue or luminance, affected the response. Anikin & Johansson (2019) reported crossmodal correspondences to single tones and lightness in speed discrimination tasks. The sound stimuli used in this study were drop and shiny: low and high sound pitch, respectively. From this perspective, the results of this study were consistent with the results of previous studies where the congruency of crossmodal correspondences affected response time and proportions of the correct responses, despite the audio stimuli used in this study being environmental sounds.

Certain studies investigated the implicit association of color–sound crossmodal correspondences and reported the effect of congruency on crossmodal correspondences and explicit associations (Sun et al., 2018). In one of these studies, the participants were initially asked to remember color/sound pairs, after which they took a discrimination test where they had to answer whether the presented pair was the same as the pair they remembered. The responses were enhanced in crossmodally congruent pairs, compared to incongruent ones. Their results showed the effect of color–sound crossmodal correspondences on discrimination tasks. These results showed that crossmodal correspondence could affect color–sound discrimination both implicitly and explicitly.

Baier et al. (2006) reported the statistical relationship between audiovisual stimuli in the discrimination task. When stimuli with a statistical relationship with audiovisual stimuli were presented, nontarget stimuli activated the relevant brain area, which was suppressed when stimuli were presented randomly. The previous study used the angle of the visual stimulus and sounds as the stimuli associated with crossmodal correspondences. The study indicated that the statistical relationship between stimuli was used in discrimination even when they were not semantically connected. It was verified that the crossmodal correspondences are generated from the statistical associations in daily life (Spence, 2011). With daily learning through a statistical relationship, the associations between color and sound may be processed automatically. The interference or enhancement of the classification process, as observed in our results, could therefore be a result of this automatic processing on crossmodal correspondences.

One of the limitations of this experiment was that there was no control condition in the sound discrimination task, making comparison to the control condition impossible. This also meant that the number of conditions for tasks differed, necessitating multiple tests. This problem was solved in Experiment 2 and subsequent experiments by using only the chromatic condition and no visual stimuli as the control condition.

The semantic and/or perceptual process involved in this interference is unclear. We could not assess whether this interference occurs at the semantic or perceptual level in Experiment 1. Previous studies have discussed whether crossmodal correspondence was semantically or perceptually dependent (Martino & Marks, 1999; Vallet et al., 2010). Some studies insisted that the Stroop effect occurred through semantic information competing (Kinoshita et al., 2018; Klein, 1964; Risko et al., 2006; Roelofs, 2003). Notably, if there is a difference in perceptual processing, the cognitive processing also differs. To investigate the independent effect of cognitive processing, we must keep the perceptual processing constant while altering cognitive processing. This question was posed in Experiment 2.

## Experiment 2. Effects of Color Word–Sound Crossmodal Stroop

The purpose of this experiment was to determine whether the Stroop effect resulting from crossmodal correspondences was observed without perceptual factors. In Experiment 2, we investigated whether the Stroop effect depended on semantic factors (color category) or perceptual factors (color appearance). In Experiment 1, we confirmed the Stroop effect between color and sound using color patches. Typically, semantic conflict rather than perceptual conflict is involved in the Stroop effect (Roelofs, 2003). Based on this perspective, we hypothesized that the Stroop effect between color and sound should be caused without perceptual factors because of conflict between the crossmodal correspondence information of presented color and sound. Consequently, the Stroop effect should occur when color words are used instead of color patches because color words are semantically identical to color patches and the perceptual factors, such as the color appearance, are not contained.

### Materials and Methods

#### Participants

The sample size was determined to be the same as that of Experiment 1. Twenty adults (1 female, 19 males; age range: 22–25 years [*M* = 23.1, *SD* = 0.787]) with corrected vision and normal hearing participated in this experiment. The procedural details were explained to all participants, and they provided informed consent. The experiment was conducted after receiving approval from the Ethical Review Committee for Research Involving Human Subjects of Toyohashi University of Technology (Approval number: 2021-02).

#### Stimuli and Apparatus

**Figure 6.**
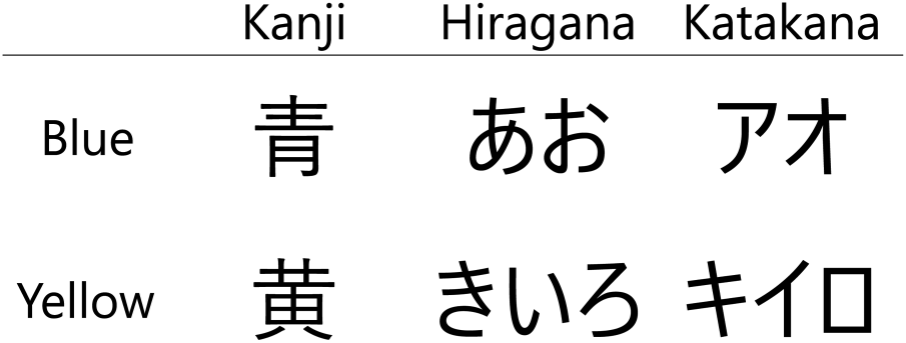
Color words as the visual stimuli (Experiment 2). Japanese has three types of characters: kanji, hiragana, and katakana. These have the same pronunciation: /ao/ for blue and /Kiiro/ for yellow, but different shapes. To prevent the participant from distinguishing the words based on the shape of the letter, these three types of characters were used in this experiment.

Visual and auditory stimuli were used in this experiment. For the visual stimuli, Japanese words representing blue and yellow were prepared. These stimuli consisted of the three semantically identical types of letters (hiragana, katakana, and kanji) for six types of visual stimuli in total. They were used to prevent the participant from solely basing their identification on the shape of characters and not the meaning of the word. All characters were presented in black and Yu Gothic UI font. The actual words used are shown in Figure 6.

The same auditory stimuli used in Experiment 1 were prepared (drop and shiny). The loudness of these sound stimuli was controlled to 80 phon. The experiment’s environments and equipment were the same as those in Experiment 1.

#### Procedure

**Figure 7.**
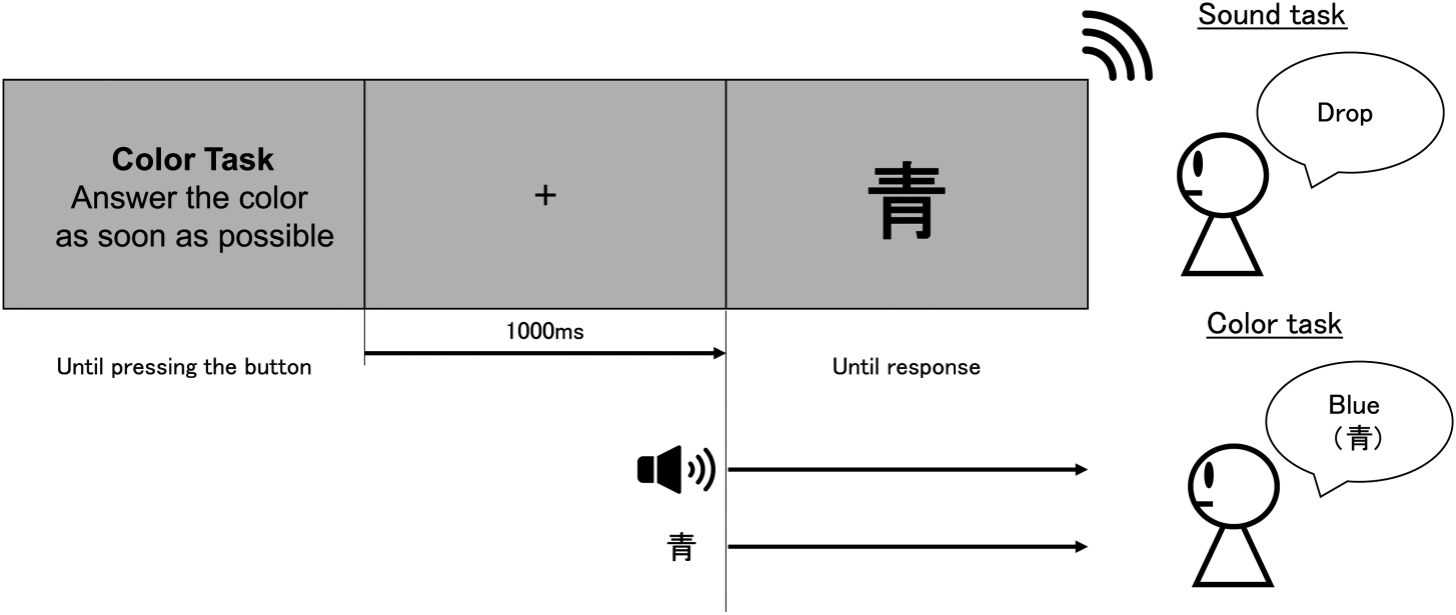
Experimental protocol for one trial (Experiment 2). The protocol was the same as the one in Experiment 1, except for the visual stimuli. The participant was required to give the meaning of the presented word instead of the color of the visual stimulus. The sound and color stimuli were presented simultaneously.

The experiment’s procedure was the same as that in Experiment 1 except that the visual stimuli differed; color words were presented instead of color patches. In addition, a control condition was added to the sound discrimination task where no visual stimuli were presented. Visual and auditory stimuli were presented simultaneously. Similar to Experiment 1, we checked the reaction time in a preliminary experiment. There was no difference in the reaction time for the control condition between the sound and color discrimination tasks when the auditory and visual stimuli were presented simultaneously. Therefore, we decided to present the auditory and visual stimuli simultaneously in Experiment 2. The experiment consisted of two tasks in the same way as in Experiment 1. However, in the color discrimination task, participants were asked to respond to the meaning of the presented words. In the sound discrimination task, two types of sound stimuli (drop/shiny) were presented, and three types of color stimuli (blue/yellow/none) were presented with 2×3=6 conditions. In the color discrimination task, there were two types of color stimuli (blue/yellow) and three types of sound stimuli (drop/shiny/none), with 2×3=6 conditions. Each participant in this experiment performed (6 conditions + 6 conditions) × 20 repetitions = 240 trials. The sound and color discrimination tasks were alternated every six to eight trials. The sounds and colors were presented in random order. To eliminate the influence of button presses, the placement of the buttons was randomized for each participant, and the button presses were unified. Participants responded to the presented color words or the sound by pressing a button. Figure 7 shows the flow of one trial.

#### Analysis

The collected data were analyzed in the same manner as in Experiment 1. The trials were separated into congruent, incongruent, and control conditions based on the pair of presented color words and sound stimuli (Table 5).

**Table 5.** Combination of stimuli and congruency conditions (Experiment 2). This congruency was determined to be the same as in Experiment 1.

Task	Sound	Color	Congruency
Sound	Drop	Blue	Congruent
	Shiny	Blue	Incongruent
	Drop	Yellow	Incongruent
	Shiny	Yellow	Congruent
	Drop	None	Control
	Shiny	None	Control
Color	Drop	Blue	Congruent
	Shiny	Blue	Incongruent
	None	Blue	Control
	Drop	Yellow	Incongruent
	Shiny	Yellow	Congruent
	None	Yellow	Control

The proportions of correct responses and response time for each condition were calculated for each participant. Response time for each participant was standardized through *z*-score normalization. An ANOVA was then performed on the distribution of the proportions of correct responses and response time for all participants.

### Results

#### The Proportions of Correct Response

**Figure 8.**
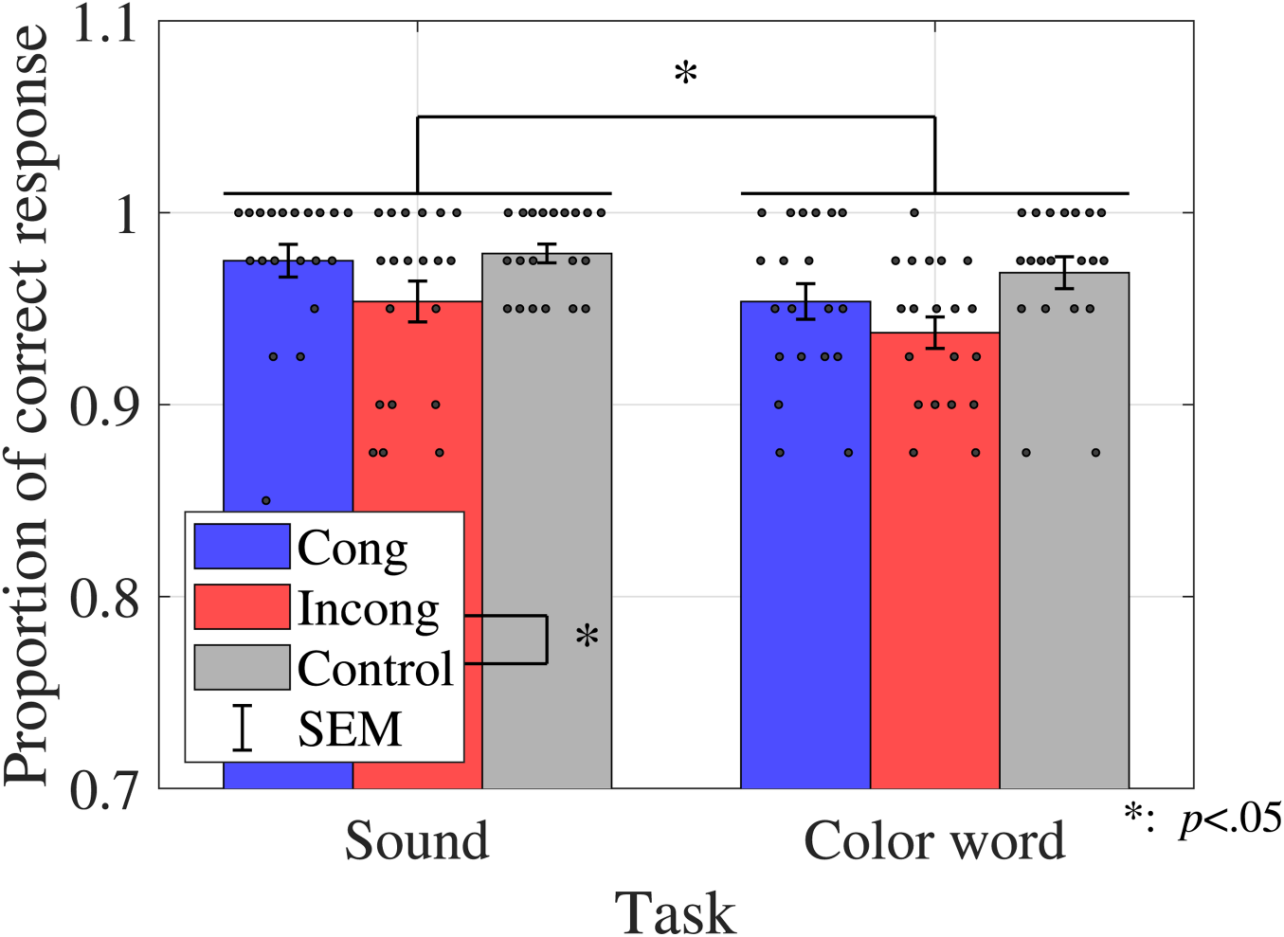
Proportions of correct responses (Experiment 2). Bar graphs and error bars show the average and standard errors of proportion of correct responses. Dots show the result for individuals (*N* = 20). Left three bars show the results for the sound discrimination task and right three bars show the results for the color discrimination task. The accuracy in the incongruent condition was lower than that in the control condition, and it was higher in sound discrimination than in color discrimination. Abbreviations: Cong = congruent; Incong = incongruent; SEM = ±1 standard error of the mean.

The average proportions of correct responses were calculated for each condition, as shown in Figure 8. The error bars indicate the ±1 standard error of the mean (SEM).

A two-way ANOVA was performed to compare the effect of congruency and task on the proportions of correct responses. The results revealed a statistically significant primary effect of the congruency ( $F(1.53,29.03)=6.9837,p=.0061,\eta_{p}^{2}=0.2688$ ) and task ( $F(1,19)=5.6267,p=.0284,\eta_{p}^{2}=0.2285$ ). The multicomparison revealed a significant difference between the incongruent and control conditions ( t(19)=4.1955,p=.0005,adj.p=.0015 ). Table 6 shows the results of the ANOVA on both the sound and color discrimination tasks.

**Table 6.** ANOVA results for the proportions of correct responses. They revealed significantly lower accuracy for the incongruent condition than the control condition and significantly higher accuracy in the sound discrimination task compared to the color discrimination task.

Main effect		*F*	*p*		* $p.eta^{2}$ *
	Congruency	6.9837	.0061	**	0.2688
	Task	5.6267	.0284	*	0.2285
	Congruency × Task	0.2957	.7331	*ns*	0.0153
Multicomparison		*t*	*p*	*adj.p*	
Congruency	Incong < Control	3.6084	.0019	.0056	*
	Cong = Incong	2.0226	.0574	.0574	*ns*
	Cong = Control	1.7260	.1006	.1006	*ns*

Abbreviations: Cong = congruent; Incong = incongruent; ANOVA = analysis of variance.

*: p<.05 ,**: p<.01 .

#### Response Time

**Figure 9.**
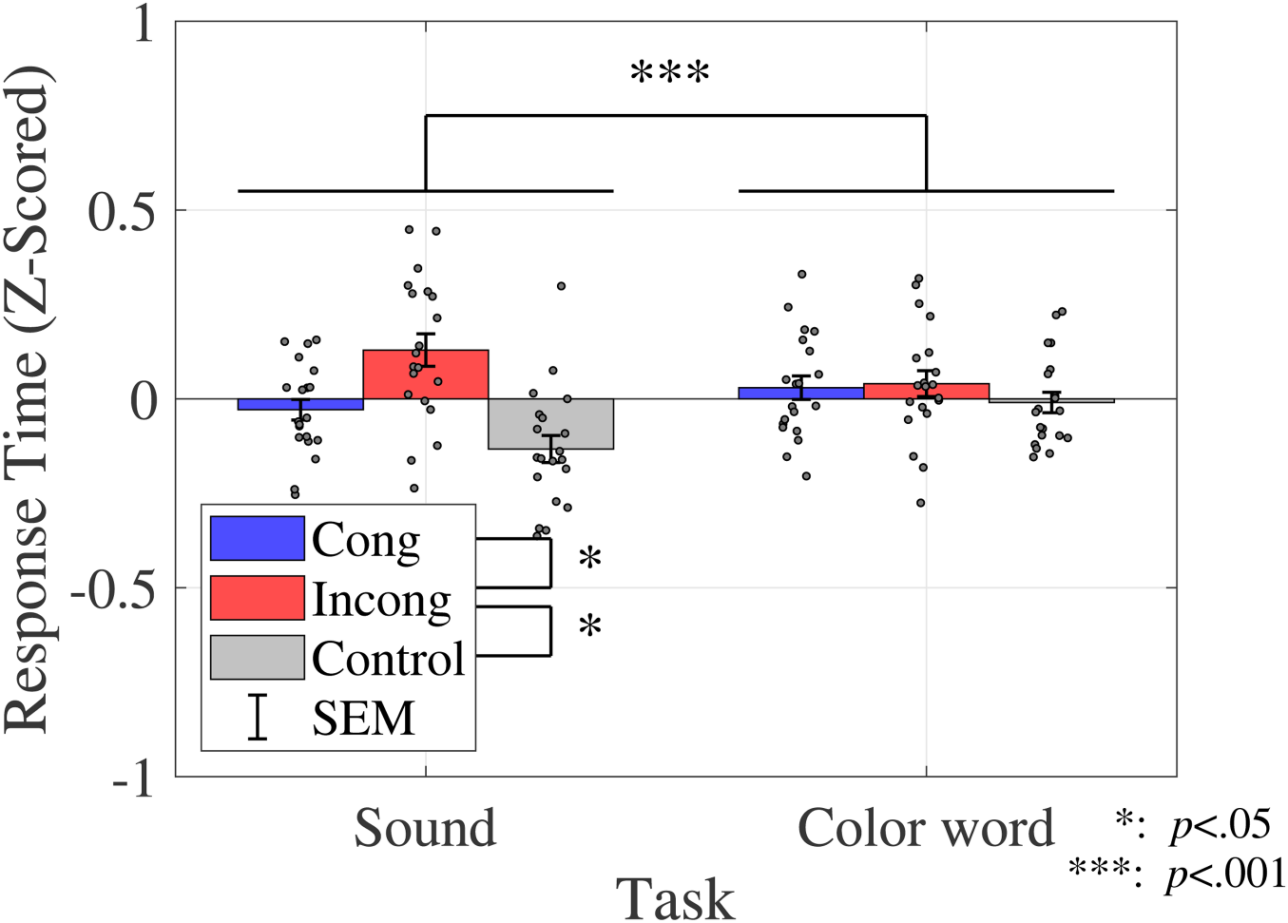
Response time (Experiment 2). Bar graphs and error bars show the average and standard errors of proportion of correct response, respectively. Dots show the result for individuals (*N* = 20). The left three bars show the sound discrimination task and the right three bars show the results for the color discrimination task. The incongruent condition yielded a longer response time than the other two conditions, whereas sound discrimination yielded a shorter response time than color discrimination. Abbreviations: Cong = congruent; Incong = incongruent; SEM = ±1 standard error of the mean.

The average response time was calculated for each condition, as shown in Figure 9.

A two-way ANOVA was performed to compare the effect of congruency and task on the response time. The results revealed a statistically significant primary effect of congruency ( $F(1.92,36.56)=9.3267,p=.0006,\eta_{p}^{2}=0.3293$ ) and task ( $F(1,19)=15.8304,p=.0008,\eta_{p}^{2}=0.4545$ ). The multicomparison revealed the significant difference between the incongruent and control conditions ( t(19)=3.9957,p=0.0008,adj.p=.0023 ) and between the congruent and incongruent conditions ( t(19)=2.5714,p=.0187,adj.p=.0187 ). Table 7 shows the results of the ANOVA for both the sound and color discrimination tasks.

**Table 7.** ANOVA results for the response time. They revealed the significantly longer response time for the incongruent task than other conditions and a significantly shorter response time for sound discrimination than color discrimination.

Main effect		*F*	*p*		* $p.eta^{2}$ *
	Congruency	9.3267	.0006	***	0.3293
	Task	15.8304	.0008	***	0.4545
	Congruency × Task	2.9872	.0762	+	0.1359
Multicomparison		*t*	*p*	*adj.p*	
congruency	Incong > Control	3.9957	.0008	.0023	*
	Cong < Incong	2.5714	.0187	.0187	*
	Cong < Control	1.9709	.0635	.0635	*ns*

Abbreviations: Cong = congruent; Incong = incongruent; ANOVA = analysis of variance.

+: p<.10 , *: p<.05 , ***: p<.001 .

### Discussion

In Experiment 2, a Stroop task involving color words and sounds was conducted to investigate whether the effect of the crossmodal correspondence was perceptually dependent (color appearance).

The results showed the Stroop effect due to color words and sound crossmodal correspondences, thereby indicating that crossmodal correspondence between color and sound could occur even when perceptual factors (color vision) were excluded from the visual stimuli. The word stimuli used in Experiment 2 were semantically identical to those used in Experiment 1 (Yamamoto, 1994, 2009). Moreover, no difference was observed in the Stroop effect among Japanese character forms (Coderre et al., 2008). These results indicate that the interference caused by crossmodal correspondence may depend on semantic factors, such as color category.

Previous studies that investigated whether crossmodal correspondence was semantically or perceptually dependent were controversial (Martino & Marks, 1999; Odgaard et al., 2003; Vallet et al., 2010). A study of crossmodal correspondence between lightness and pitch has shown that response interactions occurred even when presented with letters indicating brightness (BLACK–WHITE) and words related to brightness (NIGHT–DAY) (Martino & Marks, 1999). This was consistent with the results of this study, where interference occurred between color words and environmental sounds, thereby suggesting that interference occurred depending on the semantic information represented by the presented words. It should be noted that there have been cases reported in which perception was modulated by crossmodal correspondence (Mishra et al., 2007; Vallet et al., 2010). The experiments investigating crossmodal correspondences using a priming task have indicated perceptual dependence (Vallet et al., 2010). Such experiment tasks involved discriminating visual or auditory stimuli those were learned as a pair in the learning phase. It was shown that interference was not observed when visual stimuli were presented with sound that was not presented as a pair during the learning phase. This result indicated that the crossmodal correspondence effect depended on the input modality. Previous studies have focused on the formation of crossmodal correspondence during the learning phase of the priming task, whereas, as the present results showed, the effect of the already associated stimuli may not solely be perceptually dependent.

The present results were also consistent with those of studies showing that the typical Stroop effect caused conflicts in semantic relatedness (Augustinova & Ferrand, 2012; Kinoshita et al., 2018; Klein, 1964; Risko et al., 2006; Roelofs, 2003). A study investigating the semantic relevance of words in the Stroop effect reported that the degree of interference in the Stroop effect varied depending on the semantic gradient and that the degree of interference was determined by whether the word was semantically related to whether it represented a color or not (Klein, 1964). Therefore, the semantic Stroop effect should be caused by competing semantic information of the word. In this study, the competition for the crossmodal correspondence of the color category may have caused the interference of the response.

The pronunciation of Japanese words might affect the results of this study. We used blue and yellow translated in Japanese, Ao [/ao/] and Kiiro [/kiiro/], with different character types but the same pronunciation. Miyahara et al. (2012) studied Japanese vowel and color–sound crossmodal correspondences. The color and vowels were found to be associated; blue was associated with /o/ sound and yellow with /i/ sound. Moreover, low-pitched sound enhanced the blue-/o/ association, and high-pitched sound enhanced the yellow-/i/ association. According to their study, there might be the mediation of pronunciation and not the mediation of semantic information. However, in our experiment, the participants were not required to pronounce the word during the tasks. Therefore, the mediation of semantic information should be investigated in future studies.

## Experiment 3. Color Categorizing Modulation With Crossmodal Correspondences

The results of Experiments 1 and 2 indicated that crossmodal Stroop interference could be caused by semantic conflict. An earlier study showed that color categorization was influenced by contextual factors. It has been reported that when intermediate colors, which could be labeled as either of two colors with a probability of 50% each, were used and the color of the letter or figure were responded to, the response rate was affected depending on the meaning of the letter or figure (Kubat et al., 2009). The results indicated that the color categorization modulated the effect of crossmodal correspondence depending on the meaning of the letter or figure, which suggested that semantic processing may affect the color classification through a top-down process by automatically performing semantic processing. Based on the previous study, there is a possibility that the color classification process on the Stroop effect is affected by crossmodal correspondence. To investigate the way in which color classification was affected by crossmodal correspondence, we examined the boundary of two color classifications when listening to a sound. If the crossmodal correspondence affected the color classification, the boundary should be affected by the congruency of crossmodal correspondence, and the range of color presented with correspondence sound would increase. Gallace & Spence (2006) investigated the congruency effect of crossmodal correspondences through objective relationships and revealed semantic dependence. However, the impact of subjective labeling on crossmodal correspondence remained unclear. To investigate the impact of subjective labeling, the responses were divided into two groups (target color/nontarget color) based on the participant’s color labeling and compared. To determine the qualitative or quantitative characteristics of stimuli that affect crossmodal correspondence, investigating the behavior of crossmodal correspondence around the categorical boundary is important because hue perception is categorized as a qualitative dimension.

### Materials and Methods

#### Participants

Similar to Experiments 1 and 2, we calculated the sample size. Twenty adult males (age range: 21–25 years [*M* = 23.2, *SD* = 1.39]) with appropriate blue and yellow discrimination ability or corrected vision and normal hearing, participated in this experiment. All participants were briefed on the procedural details. Informed consent was obtained from all the participants. The experiment was conducted after receiving approval from the Ethical Review Committee for Research Involving Human Subjects of Toyohashi University of Technology (Approval number: 2021-02).

#### Stimuli and Apparatus

**Figure 10.**
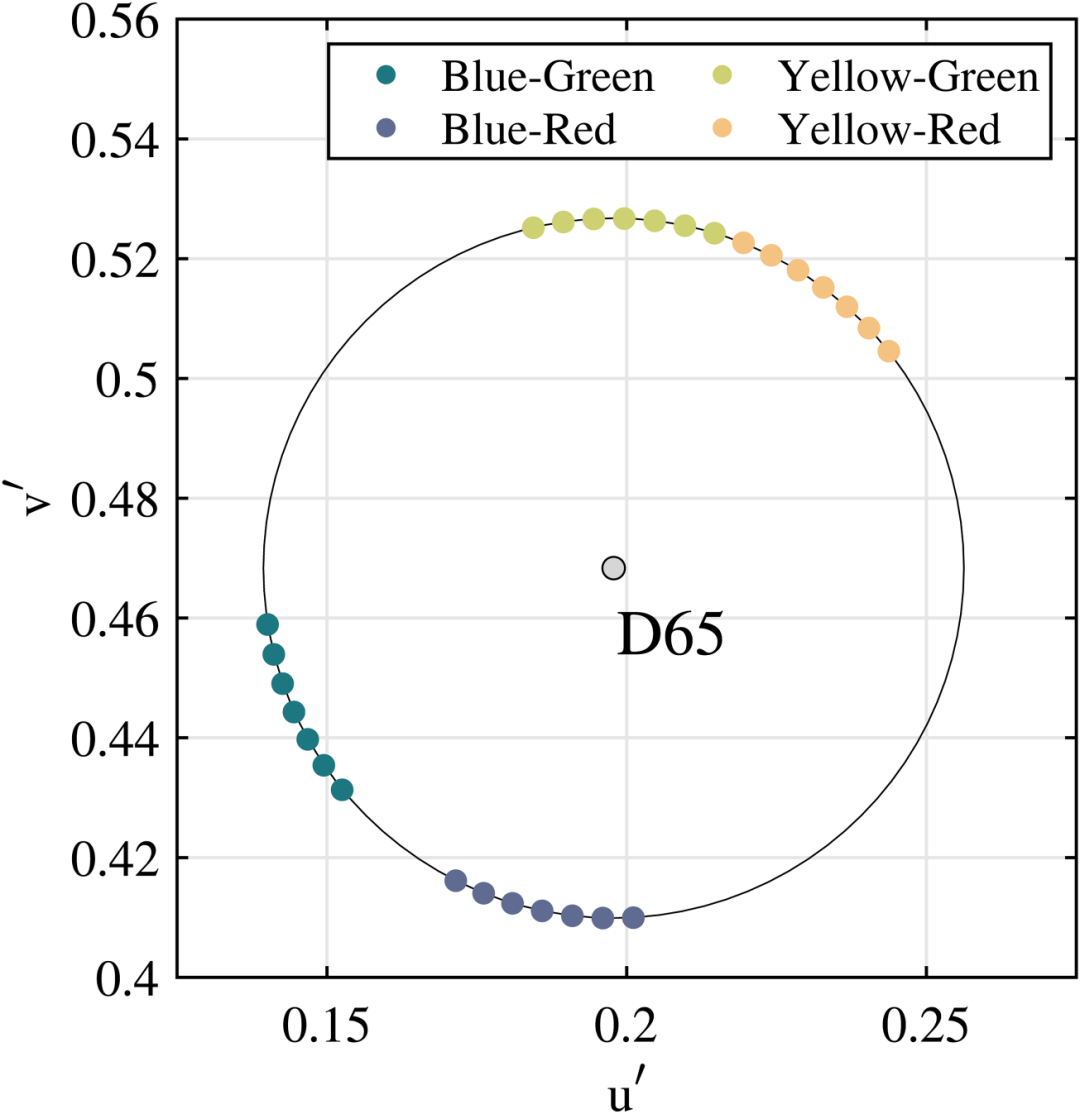
Color stimuli and color circles in the u’v’ chromaticity diagram. Those were determined based on the color category boundaries obtained in the preliminary experiment. From its boundary, seven colors were determined, which rotated from $-15^{\circ }$ to $+15^{\circ }$ in 5 ${}^{\circ }$ steps.

In a preliminary test, we estimated the ranges for blue and yellow in the hue circle with the same saturation and lightness defined on the u’v’ chromaticity diagram. Consequently, four end points (both ends for blue and yellow, respectively) were obtained. Regarding two end points for blue, one of them was assigned as the value at 0 ${}^{\circ }$ for Blue–Red, and another was assigned as the value at 0 ${}^{\circ }$ for Blue–Green. Similar to the blue, one of the end points for yellow was assigned as the value at 0 ${}^{\circ }$ for Yellow–Red, and another as the value at 0 ${}^{\circ }$ for Yellow–Green. Subsequently, the value of hue for each end point was varied in both directions on the u’v’ chromaticity diagram by 5 ${}^{\circ }$ . Consequently, seven points of hue were prepared for each of the four color pairs. Here, as the number of degrees increased, the probability of blue or yellow response increased. For example, for yellow–red, when the values were increased with 0 ${}^{\circ }$ as the center, the probability of yellow judgment was increased. The coordinates are shown in Figure 10 and Table 8. Notably, regarding prepared color stimuli, the distribution of blue was wider in the u ′ v ′ chromaticity diagram than that of yellow, as shown in Figure 10. We focused on whether the color boundary was affected by the sound stimuli that corresponded with one of the colors of the pair. Therefore, the difference in the distributions of blue and yellow was not considered in this study.

**Table 8.** Coordinates of color stimuli (Experiment 3). Each RGB value was the value at 0 ${}^{\circ }$ for each pair of colors. The RGB values were used to present the color stimuli on Psychtoolbox-3.

Color pair		RGB			Yu’v’	
Blue–Red	0.3773	0.4178	0.5735	0.1500	0.1859	0.4111
Blue–Green	0.1140	0.4666	0.5007	0.1500	0.1445	0.4443
Yellow–Red	0.9592	0.7651	0.5049	0.6000	0.2328	0.5152
Yellow–Green	0.8050	0.8210	0.4457	0.6000	0.1996	0.5267
Background	0.8500	0.8500	0.8500			

**Figure 11.**
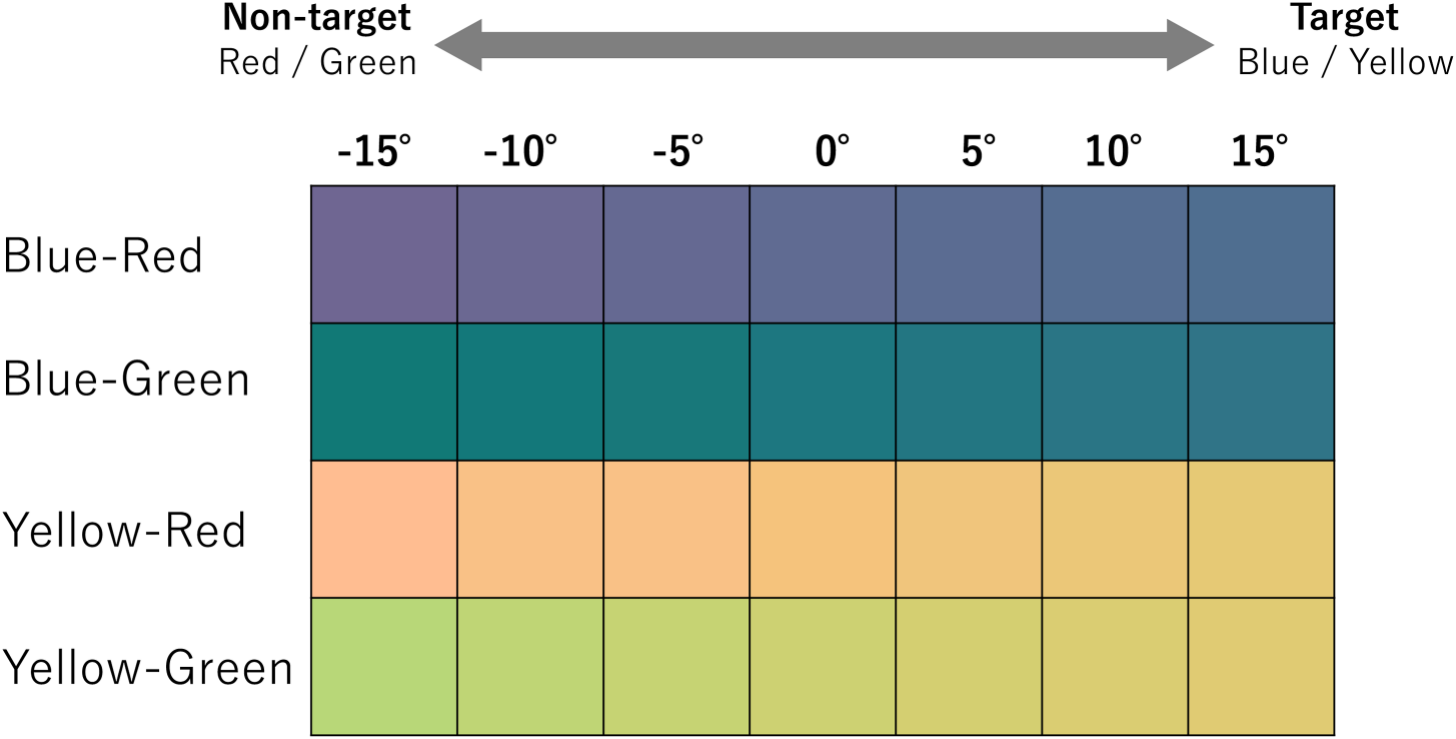
Color stimuli used in Experiment 3. The color at 0 ${}^{\circ }$ indicates the end points of blue or yellow based on the results of the preliminary test. For each pair of colors (Blue–Red, Blue–Green, Yellow–Red, and Yellow–Green), the values of hue were varied in both directions on the u’v’ chromaticity diagram by 5 ${}^{\circ }$ .

This experiment constituted four sessions to obtain the color boundaries. In each session, seven levels of color were prepared, and thus, there were 28 colors in total. The visual stimuli used in this experiment are shown in Figure 11. The *x*-axis shows the relative hue angle from the end point (at 0 ${}^{\circ }$ ) of blue and yellow determined based on the results of the preliminary experiment.

The auditory stimuli used were similar to those used in Experiments 1 and 2. The loudness of these sound stimuli was set to 80 phon. The experiment environments and equipment were the same as those in Experiments 1 and 2.

#### Procedure

**Figure 12.**
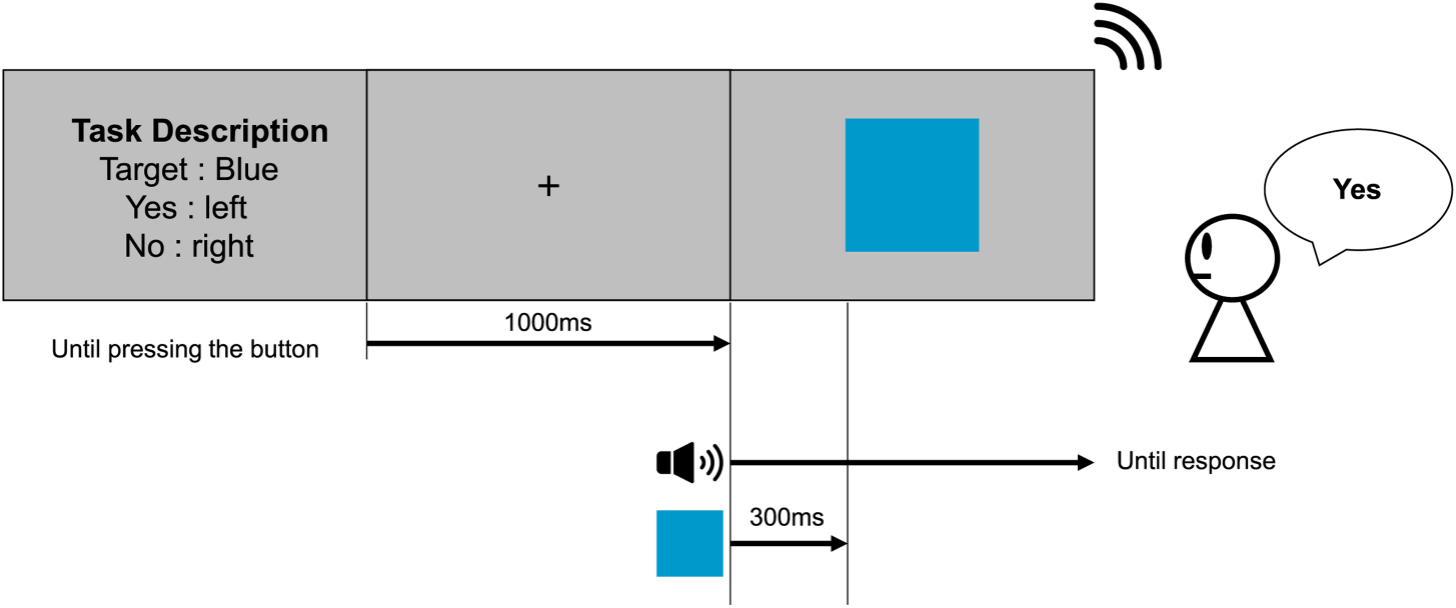
Experimental protocol for one trial (Experiment 3). Participants were required to answer whether the presented color was labeled as the target color (blue or yellow). The color stimuli were presented only for 300 ms. The sound stimuli were presented until the participant’s response was confirmed.

At the beginning of the experiment, the auditory stimuli were presented and confirmed by the participants. Next, the instructions for the experiment were presented on the screen. After the fixation cross was presented for 1,000 ms, the visual and auditory stimuli were presented simultaneously. We did not conduct a preliminary experiment because only the visual task was implemented. Similar to Experiment 2, we then decided to present the auditory and visual stimuli simultaneously. The visual stimuli were presented for 300 ms, after which they disappeared from the screen. To avoid the effect of chromatic adaptation on the judgment in a subsequent trial, we decided that the visual stimuli should disappear after 300 ms of presentation. After the visual stimuli disappeared, the participants stated whether they thought the presented color could be labeled as the target color (blue/yellow) by pressing a button corresponding to yes or no. The auditory stimuli were replayed until a response was given. There were four sessions, where the stimuli were presented randomly from seven colors in the color pairs (Blue–Red/Blue–Green/Yellow–Red/Yellow–Green). The sound variable consisted of two different sound conditions (drop/shiny) and a control condition of silence (none), which were presented randomly. Participants performed four sessions × 3 sounds × 7 hue angles × 5 repetitions = 420 trials. The flow of one trial is shown in Figure 12.

#### Analysis

Similar to Experiments 1 and 2, MATLAB R2018b (MathWorks) and R software (Version 4.2.0) were used for analyses. The trials were categorized into congruent, incongruent, and control conditions based on the pair of presented target color and sound stimuli (Table 9).

**Table 9.** Combination of stimuli and congruency conditions (Experiment 3). Those were determined in the same manner as in Experiments 1 and 2.

Target color	Sound	Congruency
Blue	Drop	Congruent
	Shiny	Incongruent
	None	Control
Yellow	Drop	Incongruent
	Shiny	Congruent
	None	Control

**Figure 13.**
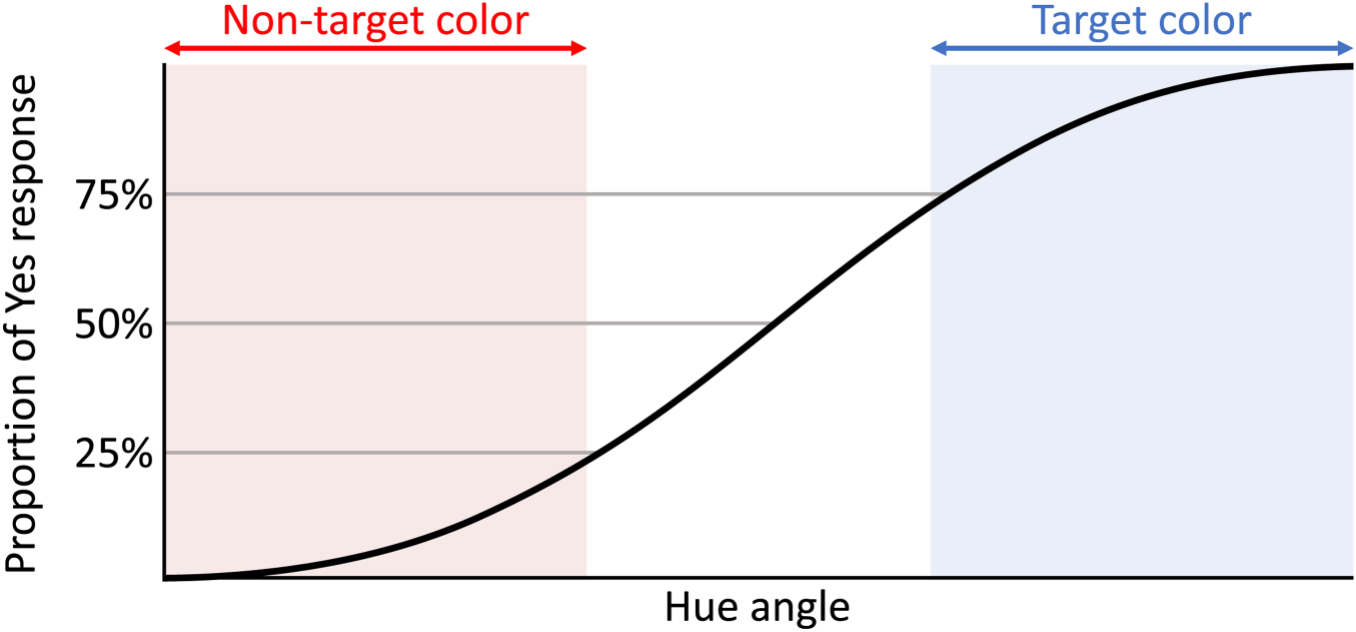
Target/nontarget trials classification based on the psycho-metric curve. Based on the hue angle on 75% and 25% of yes response probability, trials were separated into two groups; target and nontarget color categories.

For each hue angle and each congruency condition, the proportion of yes responses was calculated. Based on these values, the PSE (Fechner et al., 1966; Pelli & Bex, 2013) of each participant was calculated from the psychometric function obtained through logistic regression using MATLAB. For the response time analysis, the responses were separated based on the congruency and color category boundary (Figure 13). Target color category boundary was defined as the hue angle at which the probability of yes responses was 75%. Trials using colors with hue angles larger than this boundary were defined as target color trials. Nontarget color category boundary was defined as the hue angle at which the probability of yes responses was 25%. Trials using colors with hue angles smaller than this boundary were defined as nontarget color trials. The response time of each condition (target and nontarget color category conditions) was calculated for each participant. Response time was standardized for each participant using *z*-score normalization. An ANOVA was then performed on the distribution of PSE and the response time for all participants.

### Results

#### Point of Subjective Equality

**Figure 14.**
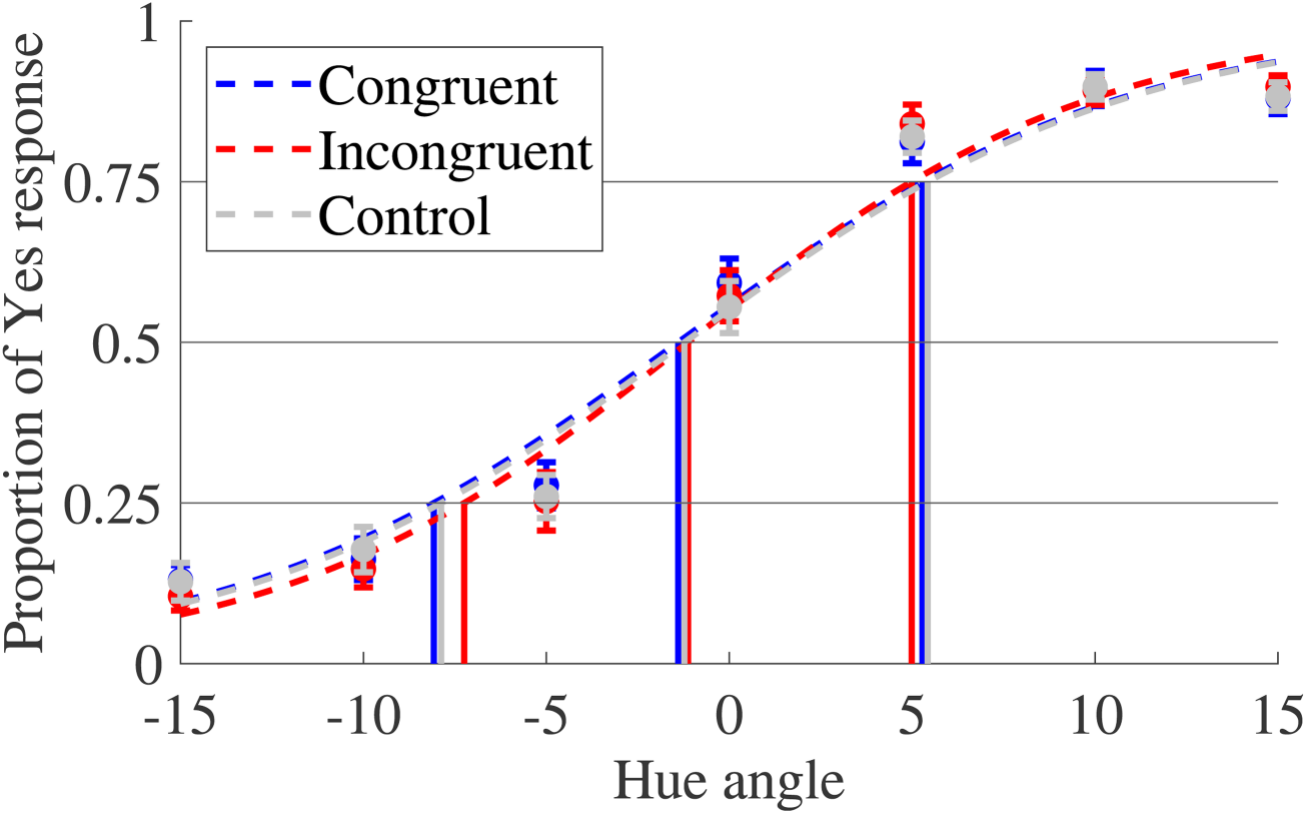
Average and standard errors of proportions of yes responses and estimated psycho-physical curves. Each plot shows the average proportion of yes responses. Based on these plots, the psycho-metric curves were estimated as shown in dotted lines. From these lines, the hue angles on 75%, 50% (point of subjective equality [PSE]), and 25% of yes response proportions were obtained.

From the proportion of yes responses of each participant to each condition, the average proportion of yes responses, standard deviation for all participants, and the estimated psycho-physical curves were calculated. The average psycho-physical curves are shown in Figure 14.

**Figure 15.**
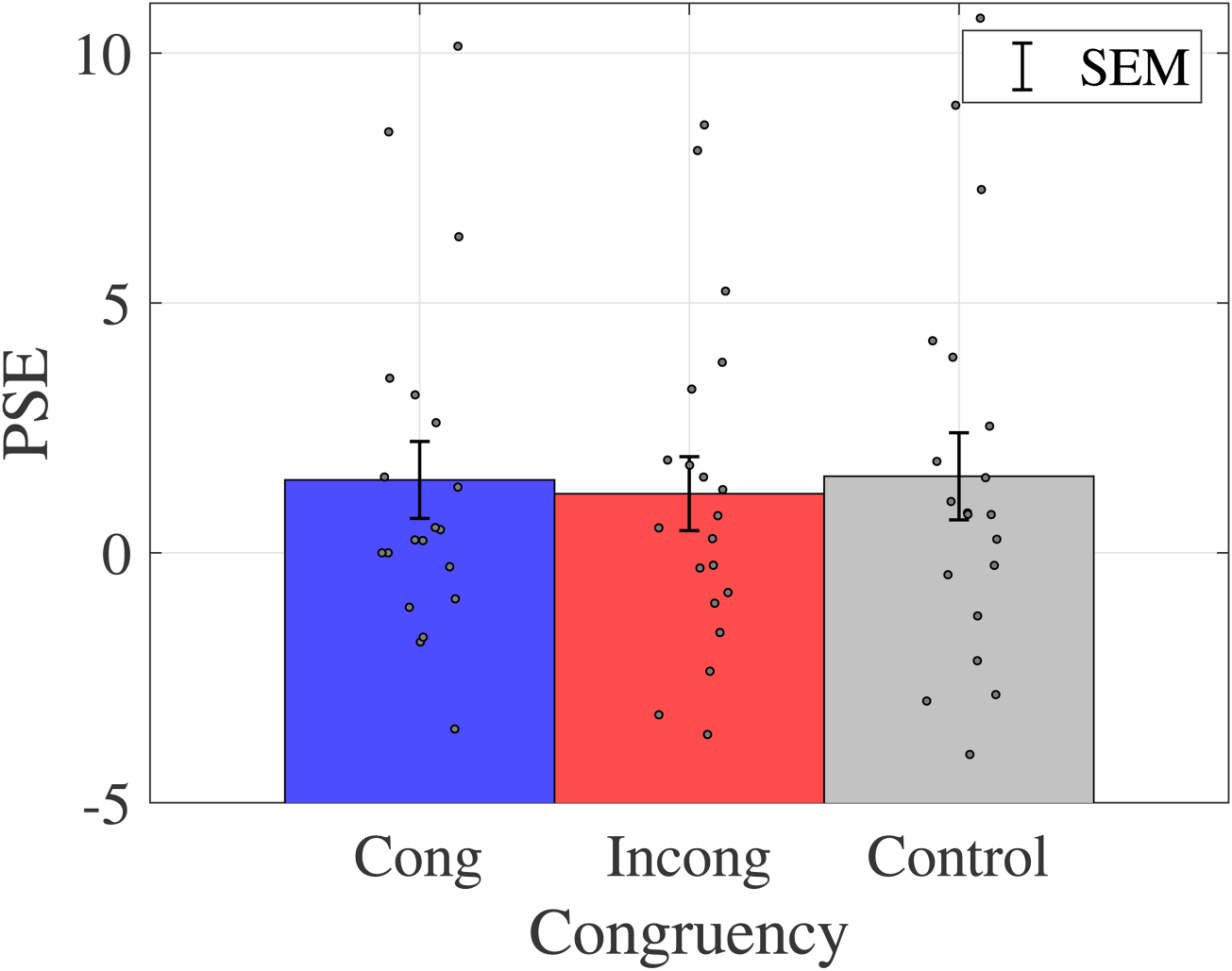
Average and standard error of PSE for each participant. Bar graphs and error bars show the average and standard errors of PSE, respectively. Dots show the result for individuals (N = 20). No significant difference was observed among those conditions. Abbreviations: Cong = congruent; Incong = incongruent; SEM = ±1 standard error of the mean; PSE = point of subjective equality.

The within-participant means and standard deviations for the PSE are shown in Figure 15.

A one-way ANOVA was conducted to examine the effect of congruency on color classification boundary, and there was no main effect of congruency on PSE ( $F(2,19)=0.5421,p=.5834,\eta_{p}^{2}=0.0277$ ).

#### The Average of Response Time

**Figure 16.**
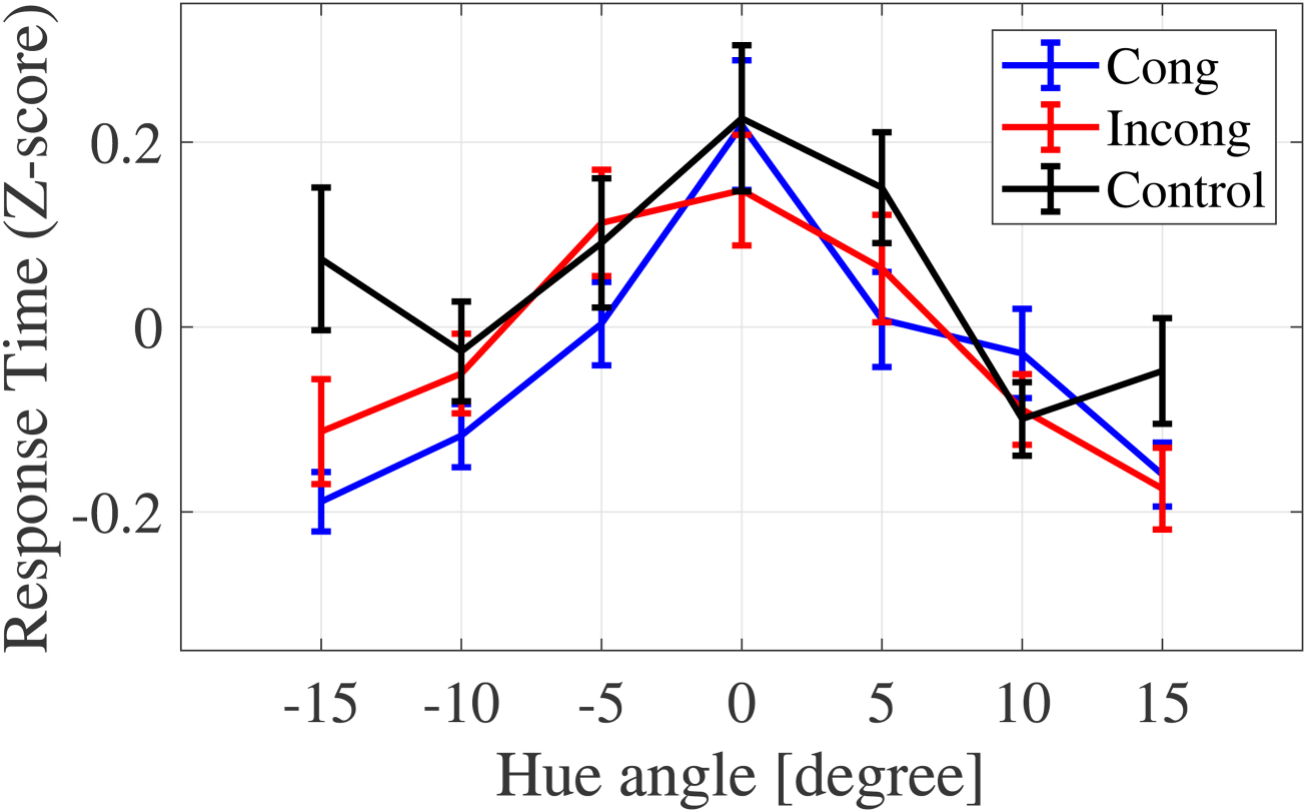
The average response time for each condition. Response time differs depending on the congruency and color category conditions.

The average response time for each condition is shown in Figure 16.

**Figure 17.**
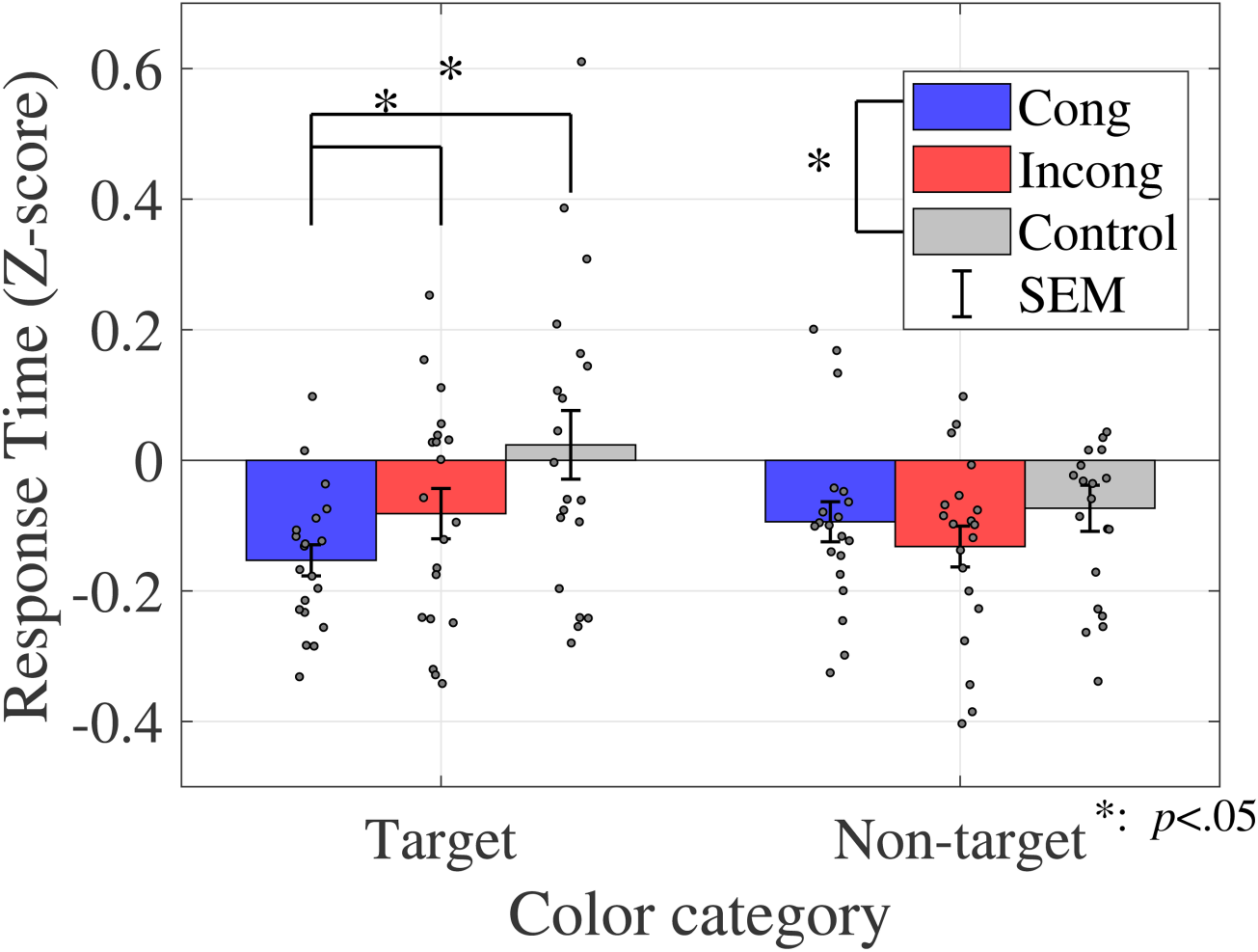
Average response time of target/nontarget color category conditions. The congruent condition yielded a shorter response time than the control condition. For the target condition, the interaction yielded a shorter response for the congruent condition than the other two conditions.

To compare the Stroop effect by color category, the data were divided into two groups (target/nontarget color categories) based on the hue angle, where the yes probabilities were 75% or 25% on the psycho-metric curve (Figure 13). The averages of response time were calculated for target and nontarget color category conditions, respectively (Figure 17).

A two-way ANOVA revealed a statistically significant main effect of the congruency ( $F(1.49,28.24)=4.6921,p=.0259,\eta_{p}^{2}=0.1980$ ) and the interaction between the congruency and category ( $F(1.84,34.98)=4.5121,p=.0204,\eta_{p}^{2}=0.1919$ ) (Table 10). According to the multicomparison, a significant difference was observed between the congruent and control conditions ( t(19)=2.9600,p=.0080,adj.p=.0241 ). For the target color category condition, there were significant differences between the congruent and control conditions ( t(19)=3.5966,p=.0019,adj.p=.0058 ) and between the congruent and incongruent conditions ( t(19)=2.2005,p=.0403,adj.p=.0403 ). Conversely, there were no significant differences for the nontarget color category condition ( $F(1.79,33.99)=1.1317,p=.3291,\eta_{p}^{2}=0.0562$ ).

**Table 10.** ANOVA results for the response time in the color categorization task. This revealed the main effect of the congruency and interaction between the congruency and the color category. This indicates the shorter response time for the congruent condition than the control condition and the other two conditions for the target color category condition.

Main effect		*F*	*p*		* $p.eta^{2}$ *
	Congruency	4.6921	.0259	*	0.1980
	Category	0.5669	.4607	*ns*	0.0290
	Congruency × Category	4.5121	.0204	*	0.1919
Interaction	Category at Cong	1.8100	.1943	*ns*	0.0870
	Category at Incong	1.0891	.3098	*ns*	0.0542
	Category at control	2.9670	.1012	*ns*	0.1351
	Congruency at target	7.0496	.0061	**	0.2706
	Congruency at nontarget	1.1317	.3291	*ns*	0.0562
Multicomparison		*t*	*p*	*adj.p*	
Congruency	Cong < Control	2.9600	.0080	.0241	*
	Incong = Control	1.9157	.0706	.0706	*ns*
	Cong = Incong	0.6706	.5105	.5105	*ns*
Congruency at target	Cong < Control	3.5966	.0019	.0058	*
	Cong < Incong	2.2005	.0403	.0403	*
	Incong = Control	1.8445	.0808	.0808	*ns*

Abbreviations: Cong = congruent; Incong = incongruent; ANOVA = analysis of variance.

*: p<.05 , **: p<.01 .

### Discussion

In Experiment 3, the effect on the boundaries of two colors by sound that was correspondent with one of the colors of the pair was examined.

The findings of PSE suggested that there was no modulation of category boundary, and crossmodal correspondence may not be used in color category judgments. Color-word Stroop effects, where the meaning of a letter modulates color category judgments (Kubat et al., 2009), have been reported. In the study, stimuli paired with a specific correct color were used (e.g., pink for flamingo). This mechanism may differ from the crossmodal correspondence that we used, and the magnitude of the interference effect may be different from the results observed in the previous study. In fact, in the Stroop effect, the interference effect between color and letter is generally asymmetric, thereby indicating that the semantic processing of words is more dominant.

The results of response time indicated that color–sound crossmodal correspondences were observed, not only for the specific colors, but also the colors labeled as the sound-associated colors. Given that the effect of the Stroop effect corresponds to the color labeling difference, subjective labeling is considered to play a role in crossmodal correspondences.

A study on the reverse Stroop effect showed that the Stroop interference rate and color categorization are affected by spectral color shifts (Smithson et al., 2006). As for the relationship between language and perception, linguistic relativity has been studied, and one hypothesis, the Sapir–Whorf hypothesis, proposed that humans perceive the world through language (Kay & Kempton, 1984). Moreover, color recognition is based on color categories and not color appearance (Berlin & Kay, 1991; Gilbert et al., 2006; Roberson et al., 2006; Uchikawa et al., 1998). When people learn crossmodal correspondence between color and sound, they learn the correspondence between the color category and the sound rather than between the color appearance and the sound. Based on this idea, the crossmodal correspondence observed in this study might be category-dependent rather than perception-dependent.

## General Discussion

This study aimed to clarify the ways in which semantic and perceptual processing relate to color–sound crossmodal correspondences. In Experiment 1, we observed that the performance of the discrimination task was more optimized for the congruent condition than for the incongruent condition, thereby indicating that crossmodal correspondence between color and sound occurred. In Experiment 2, we investigated the Stroop effect due to crossmodal correspondence between color words and sounds. The results showed that the performance of the Stroop effect differed between the incongruent and control conditions, showing that crossmodal correspondence could be affected even when perceptual factors were excluded from the stimuli. In Experiment 3, the effect of crossmodal correspondence for the categorization was investigated. Regarding the response time, the Stroop effect was observed only in the target color category condition. This indicated that qualitative rather than quantitative features of color perception play a role in color–sound crossmodal correspondence.

### Crossmodal Correspondences on Discrimination

In Experiments 1, 2, and 3, the proportion of the correct responses and response time were affected by the congruency between color and sound, particularly between hue and environmental sound, when presented simultaneously. These results indicate that the congruency of crossmodal correspondences interacts with the discrimination task. Anikin & Johansson (2019) reported the modifications in the response time and proportion of correct responses due to color and pitch congruency. Furthermore, modulations in response time have been reported not only in the crossmodal correspondence between color and sound, but also in the correspondence between color and words related to temperature (Ho et al., 2014). The present results and those from previous studies suggest that crossmodal correspondence between color and sound affects discrimination performance. This implies that people constantly discriminate objects using information from multiple modalities, such as crossmodal correspondence. In this study, we used the color–sound crossmodal correspondences, although colors have no polarity attributes. This indicates that the crossmodal correspondence affected the discrimination for the polarity of the continuous stimuli (e.g., the higher sound and the smaller size) as well as the categorical objects, such as color. Those associations might be learnt from the statistical associations obtained in the course of daily life (Spence, 2011). It has been reported that the presentation of high frequency audiovisual pairs activate brain regions, not only in the target modality, but also in the modality of the nontarget stimulus (Baier et al., 2006). A previous study insisted that statistical frequency could associate stimuli, even if they are not semantically identical to each other. This indicates that statistical relatedness is retained between stimuli and that this relatedness is used in discrimination with both modalities. Summarily, crossmodal correspondences are used to decipher the statistical relationship between objects when people discriminate an object.

As one of the limitations of this study, most participants were male (only one female participated). This is reflected in the recruit environment. Although we could not investigate the impact of this unbalanced gender ratio in this study, females have greater expressivity than males (Kring & Gordon, 1998). Based on the fact that the color–sound crossmodal correspondences involve an emotional process, female participants may yield different results. The effect of gender on color–sound crossmodal correspondence requires further research. There is still a lot to learn about the existence of color–sound associations between other colors and sounds as well as the cause of the Stroop effect observed in our results. The general rule of color–sound associations and involvement of structural, semantic, or statistical relationships on color–sound crossmodal correspondences need to be studied further.

### Semantic Factor on Crossmodal Correspondences

In Experiments 1 and 2, color patches and words representing colors were used as the visual stimuli, which were semantically identical. The response interactions were observed in both experiments. This suggests that perceptual factors were not necessary for interference in discrimination, and that semantic factors were necessary in this study.^1^ Therefore, the interaction at a higher (or later) level of processing could affect the discrimination task on color–sound crossmodal correspondences which is consistent with the results obtained from lexical crossmodal correspondences (Melara & Marks, 1990; Walker, 2012). The color–sound crossmodal correspondences may belong to the lexical crossmodal correspondences.

In Experiment 3, interference only confirmed the target color category condition, indicating that even when presenting ambiguous colors, interference occurred when the observer labeled the color associated with the sound. A previous study on crossmodal correspondence between brightness and pitch showed that response enhancement/interference occurred even when presented with letters and words related to brightness (Martino & Marks, 1999). From this perspective, crossmodal interaction may be caused by semantic information, such as color category, but not color appearance. The use of the metathetic stimulus in our study, may explain the relative modulation by the stimulus set on crossmodal correspondences. Anikin & Johansson (2019) reported on the relationship between saturation and pitch in color–sound crossmodal correspondences. In their study, the results showed the relative modulation of crossmodal correspondences using two different ranges of pitch. This relative difference might be affected by subjective labeling as qualitative properties change. In other words, the subject judging whether the sound is low or high within the stimulus, regardless of the absolute pitch used, may form a correspondence. This linguistic labeling mediation is related to the category dependence of color perception. Regarding color labeling, the Sapir–Whorf hypothesis claims that the people’s world view is influenced or determined by their native language (Kay & Kempton, 1984).^2^ To support this hypothesis, color perception has been found to depend on linguistic color categories (Gilbert et al., 2006; Roberson et al., 2006), and people recognize a color by categorizing almost 11 colors (Berlin & Kay, 1991; Uchikawa et al., 1998). Based on these results, people may recognize a color based on its linguistic color category. As a result of recognizing the color category, the crossmodal correspondences may be learned as a category-dependent association. This linguistic color categorization might affect the learning process of crossmodal correspondences and cause category-dependent interference on the discrimination task. Notably, the perceptual modulation by crossmodal correspondence has also been reported (Mishra et al., 2007; Vallet et al., 2010). To address this, we must compare the semantic and perceptual factors directly.

It is also necessary to investigate whether category dependence is a color-specific property or a general property of crossmodal correspondence. In this study, the Stroop effect differed between target and nontarget color categories owing to the congruence of crossmodal correspondence. Based on studies of color categorical perception (Berlin & Kay, 1991; Gilbert et al., 2006; Roberson et al., 2006; Uchikawa et al., 1998), people perceive colors by category rather than by color appearance. This indicates that crossmodal correspondences are category dependent. Based on the Sapir–Whorf hypothesis, which is based on the hypothesis of color categorical perception, people might recognize the world through linguistic labels. Such categorical recognition might cause category dependence for the crossmodal correspondences of other objects. The general effect of the category dependence of crossmodal correspondence could be obtained by conducting experiments using stimuli that are not colors and by comparing the Stroop effect of the target and nontarget categories.

### Role of Qualitative Characteristics in Crossmodal Correspondence

In Experiment 3, the congruency affected the reaction time for the target color category but not the nontarget color category. If quantitative characteristics of luminance affected the reaction time, the congruency effect should be observed in both color category conditions. In other words, on the graph representing the reaction time in 5-degree increments (Figure 16), it seems to be symmetry centered at 0 ${}^{\circ }$ . However, this result was not obtained. Therefore, the results of this study may support the idea that the qualitative characteristics of the perceived color affected the congruency effect. The quantitative or qualitative characteristics of stimuli that contribute to crossmodal correspondence have been discussed. Our results suggest that qualitative characteristics of visual stimuli are involved in color–sound crossmodal correspondence, particularly in color discrimination target and nontarget color boundaries.

### Conclusion

This study investigated the influence of crossmodal correspondence between color and sound on discrimination tasks to clarify the impact of crossmodal correspondences on audiovisual information integration. Through a series of experiments, the response interactions were observed depending on the congruency for the crossmodal correspondence, indicating the influence of crossmodal correspondence on discrimination. An earlier study reported that statistical relevance activates brain regions corresponding to nontarget stimuli, thereby suggesting that statistical relevance may also be used in discrimination tasks through crossmodal correspondence (Baier et al., 2006). In addition, the Stroop effect was found both in the experiments using color patches and those using color words, and the responses only modulated the target color category condition. These results indicated that color appearance was not necessary for interference, and that color categories were important. Therefore, semantic rather than perceptual factors may influence crossmodal correspondence in the enhancement/interference effect. This might be because people recognize objects depending on linguistic labels. As a result, crossmodal correspondence might be generated between categories and not between perceptual inputs.

## Notes

1. Here, perceptual factors indicate the color vision input from the eyes, such as color appearance, and semantic factors indicate the color categories labeled by the stimuli input from the eyes.

2. Note that there are certain negative results for the Sapir–Whorf hypothesis and the validity of this hypothesis has come into question (Franklin et al., 2005; Regier & Kay, 2009)
